# Antivirulence Strategy against *Candida albicans*: Cumin Essential Oil Attenuates Virulence and Enhances Host Immune Defense

**DOI:** 10.1021/acsomega.5c12490

**Published:** 2026-05-14

**Authors:** Nimisha Mahesh, Afia Harith, Anu R. Melge, Attumpurathu Nandini, Aravind Madhavan, Kuniyil Abhinand, Peralam Yegneswaran Prakash, Bipin G. Nair, Geetha B. Kumar, Jayalekshmi Haripriyan

**Affiliations:** † Amrita School of Biotechnology, 77649 Amrita Vishwa Vidyapeetham, Amritapuri, Clappana P O, 690525 Kollam, Kerala, India; ‡ Department of Microbiology, Kasturba Medical College, Manipal, Manipal Academy of Higher Education, 576104 Manipal, Karnataka, India

## Abstract

*Candida albicans* is a deadly fungal pathogen, particularly in immunocompromised individuals, where a simple superficial infection rapidly transforms into life-threatening systemic candidiasis. Virulence factors of *C. albicans* include the yeast-to-hyphae transition, biofilm formation, and protease production, which are crucial for establishing infection. Targeting virulence factors is a promising strategy to combat *C. albicans* infections, which can overcome the limitations of conventional treatment modalities. Essential oils are important in this regard as they are used in traditional medicine due to their antimicrobial capabilities. This study is focused on the antivirulent activity of cumin essential oil (CEO), which significantly reduced the key virulence factors of *C. albicans*, such as germ tube formation and protease production. A subinhibitory concentration (0.015% v/v) of CEO downregulated the expression of the *HWP1*, *ALS3*, and *RAS1* genes involved in regulating virulence. *In silico* studies demonstrated that major compounds of CEO interacted with key amino acid residues of secreted aspartyl proteases (Sap4, Sap5, Sap6) and heat shock protein (Hsp90), a regulator of hyphal formation. CEO significantly reduced biofilm formation of *C. albicans* in different simulated body fluids such as saliva, artificial urine, and tear fluid. Furthermore, CEO affected the host–pathogen interaction of *C. albicans* with THP-1 macrophages by increasing phagocytosis and inhibiting germ tube formation. Additionally, CEO treatment modulated the levels of pro-inflammatory and anti-inflammatory cytokines, suggesting that CEO also has immunomodulatory properties, in addition to its antivirulence activity. These results indicate that CEO could be a promising candidate for combating *C. albicans* infections.

## Graphical abstract

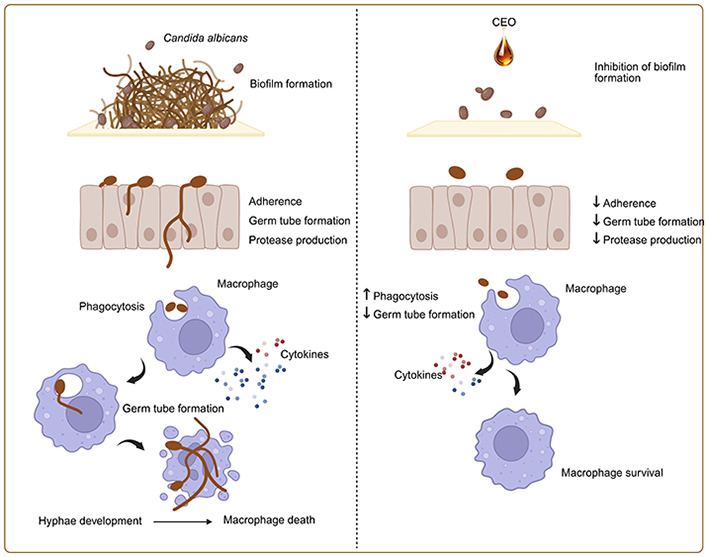

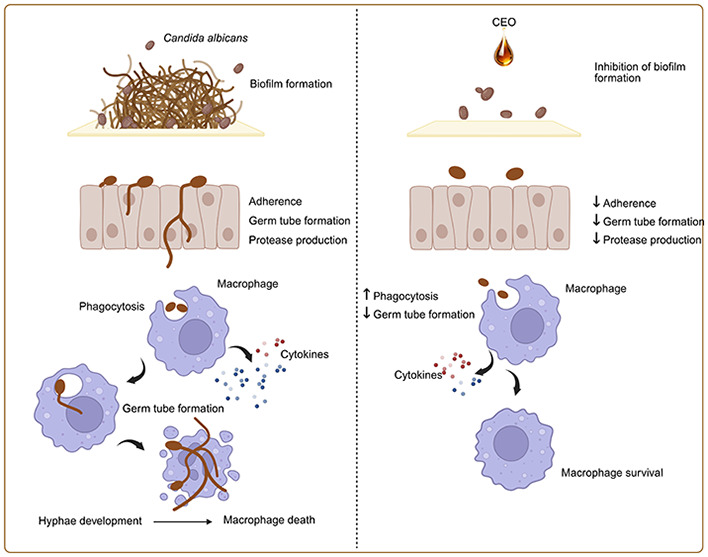

## 1 Introduction

*Candida albicans*, a yeast-like fungus, is recognized as a critical-priority pathogen by the World Health Organisation (WHO) in its first-ever fungal priority pathogen list published in 2022. It is a common microflora found in different parts of the human body, including the reproductive tract, gastrointestinal tract, and oral cavity. In immunocompromised conditions, *C. albicans* can lead to superficial and systemic infections, including oral thrush, vulvovaginal candidiasis, and life-threatening systemic infections, that cause a high mortality rate.^1^ The major pathogenic factors that allow *C. albicans* to resist antifungal therapies and persist within the host include biofilm formation, morphological switching from yeast to hyphae, secretion of proteases, and mechanisms to evade or tolerate host immune responses.^2–4^ Conventional antifungals such as azoles and echinocandins are increasingly being reduced in clinical use due to the emergence of drug resistance and significant risks of hepatotoxicity.^5,6^ This has created a strong demand for alternative therapeutic strategies that can effectively combat fungal burden and associated complications in the host. Modulation of virulence factors is a promising strategy as it primarily interferes with the pathogenicity mechanisms of the fungus without exerting fungicidal or fungistatic effects, thereby minimizing selective pressure for resistance development. This approach disarms the pathogen rather than killing it, reducing the likelihood of resistance emergence.^7^ Targeting master regulators such as the molecular chaperone Hsp90 has emerged as a promising approach as Hsp90 plays a central role in regulating *C. albicans* survival, morphogenesis, and antifungal drug resistance.^8^ Given this central regulatory role, efforts have focused on selectively modulating fungal Hsp90 function and discovery and development of species-selective Hsp90 inhibitors.^9^ Such targeting can uniquely potentiate existing antifungal agents by disrupting Hsp90-dependent virulence-associated processes, thereby offering a potential strategy to combat invasive fungal infections. However, their roles in modulating *C. albicans* virulence traits—including hyphal morphogenesis, biofilm formation, and host–pathogen immune interactions—remain understudied. Many natural compounds have been reported to attenuate microbial virulence, thereby reducing pathogen-mediated host damage without necessarily affecting microbial viability.^10^ In traditional Indian medicine, cumin essential oil is an important therapeutic agent used for the treatment of infections, ulcers, boils, cough, and inflammatory conditions.^11,12^ Cumin (*Cuminum cyminum* L.) is a widely used culinary spice and has been extensively documented for its antimicrobial, anti-inflammatory, and antioxidant activities.^13^ Previous reports have shown that cumin essential oil (CEO) possesses antibacterial and antifungal activity against clinically relevant microbes such as *Escherichia coli*, *Staphylococcus aureus,* and *C. albicans*.^15–17^ However, the impact of CEO on specific virulence factors of *C. albicans* including hyphal morphogenesis and biofilm development remains poorly understood. Therefore, the present study investigates the antivirulence potential of CEO against *C. albicans*, specifically examining its ability to attenuate critical virulence traits at subinhibitory concentrations. Unlike conventional antifungal strategies that target fungal viability, this antivirulence approach aims to disarm pathogenic mechanisms, thereby potentially reducing the selective pressure that drives antifungal resistance.^7^

Whole essential oil functions as a multicomponent system, where synergistic interactions among constituents achieve broader, more potent modulation of *C. albicans* virulence by simultaneously targeting multiple pathogenic mechanisms, surpassing the efficacy of isolated compounds. Furthermore, the immunomodulatory potential of CEO was evaluated using a macrophage coculture model to examine its effects on *C. albicans*. Through these investigations, we aim to establish CEO as a promising natural compound with both antivirulence and immunomodulatory properties for combating *C. albicans* infections.

## 2 Materials and Methods

### 2.1 Test Organism

*The C. albicans* strain (MTCC 183) used in this study was obtained from the Microbial Type Culture Collection (MTCC), Chandigarh, India. The strain was grown in potato dextrose agar (PDA) and potato dextrose broth (PDB), maintained in 80% glycerol stock and stored at −80 °C. When needed, it was revived by inoculating in PDA and incubated at 37 °C for 24 h; fresh single colonies were then inoculated into PDB and grown overnight. These overnight cultures were diluted to get an OD of 0.2 at 600 nm, which corresponded to 2.8 × 10^6^ yeast cells, and used for further studies. CEO was purchased from Bhoomi Naturals Pvt. Ltd., India. Essential oil was diluted with dimethyl sulfoxide (DMSO) at a ratio of 1:1.

### 2.2 Antimicrobial Activity—Well Diffusion Method

The Agar well diffusion method was used for checking the antimicrobial activity. The test organism *C. albicans* (MTCC-183), at an OD of 0.2 (600 nm), was evenly spread using a cotton swab to obtain a lawn culture, and wells were punctured with a diameter of 4 mm. In each well, 10 μL of essential oil was added. Amphotericin B, 10 μg/mL (Sigma-Aldrich), was used as a reference positive control, and DMSO (50%) was added as a vehicle control. The plates were incubated at 37 °C for 24 h and were observed for zone of inhibition^18^

### 2.3 Minimum Inhibitory Concentration (MIC) and Minimum Fungicidal Concentration (MBC) by the Broth Dilution Method

The MIC and MFC of CEO were determined by the broth microdilution technique. *C. albicans* suspensions were prepared in PDB and adjusted to an OD of 0.2 at 600 nm, and varying concentrations of CEO (0.5% to 0.007%) were added. Amphotericin B served as the positive control, while 0.5% DMSO was used as the vehicle control. The plates were incubated at 37 °C for 24 h, and OD was measured at 600 nm in a microtiter plate reader (Synergy HT Microplate Reader, BioTek Instruments). To determine MFC, 2 μL aliquots were spot inoculated into PDA plates and incubated at 37 °C for 24 h. The lowest concentration that inhibited visible fungal growth was recorded as MFC.^19^

### 2.4 Growth Kinetic Assay

Fungal culture adjusted to an OD of 0.2 was added (100 μL) to a sterile 96-well microtiter plate with CEO (0.5% to 0.03%) and incubated at 37 °C. OD was measured at 4, 8, 12, and 24 h.^20,21^

### 2.5 Cell Culture and Cytotoxicity Assay

THP-1 monocytes were seeded in RPMI-1640 medium with 10% fetal bovine serum (FBS) at a density of 1 × 10^4^ cells per well in 96-well plates and differentiated into macrophages by treatment with 100 nM phorbol 12-myristate 13-acetate (PMA), followed by incubation for 24 h at 37 °C in a humidified atmosphere containing 5% CO_2_. L-929 and 3T3-L1 mouse fibroblast cell lines were cultured in 96-well plates containing Dulbecco’s Modified Eagle Medium (DMEM) supplemented with 10% FBS and incubated under similar conditions for 24 h.^22^ Upon reaching approximately 80% confluency, the cells were treated with different concentrations of CEO (0.5–0.007%), along with untreated controls and 0.5% DMSO vehicle controls, and incubated for an additional 24 h. Cytotoxicity was evaluated using the MTT assay in THP-1-derived macrophages and L-929 and 3T3-L1 cells. Briefly, after treatment, 10 μL of MTT solution (5 mg/mL; 3-(4,5-dimethylthiazol-2-yl)-2,5-diphenyl tetrazolium bromide) was added to each well and incubated for 3 h at 37 °C with 5% CO_2_. The reaction was terminated by adding 100 μL of DMSO to dissolve the formazan crystals, and absorbance was measured at 570 nm using a microplate reader.^23^

### 2.6 Propidium Iodide Influx Assay

Following overnight incubation of *C. albicans* cells with CEO (0.5–0.015%) at 37 °C, the cells were washed with PBS and subsequently incubated with propidium iodide (Sigma-Aldrich; 0.5 μg mL^–1^) for 30 min at room temperature.^24,25^ Cell suspensions placed on a glass slide were examined under the fluorescent microscope (OLYMPUS TH4-200) using a TRITC filter set [excitation (535–560) nm; emission (590–650) nm]. Cells exhibiting red fluorescence were considered membrane-compromised or dead. Amphotericin B was used as a positive control.

### 2.7 Germ Tube Formation Assay

Overnight culture of *C. albicans* (OD 0.2) was further diluted to 1:1000 in PDB with 20% fetal bovine serum (FBS), and a sub-MIC concentration of CEO was added to the culture and further incubated at 37 °C. The hyphal formation was visualized under microscope at 4 and 24 h.^26^ At least 10 randomly selected microscopic fields were analyzed per condition. Hyphal length was measured from the base to the tip using ImageJ software^27^

### 2.8 Wrinkled Colony Morphology

The effect of CEO on the colony morphology was studied by incorporating sub-MIC concentrations of oil directly into the PDA; subsequently, 5 μL of *C. albicans* culture was spotted at the center of the agar surface and incubated at 37 °C. Colony morphology was examined after 5 days of incubation. Cellular morphology was evaluated, images were captured by microscopy (10X), and cells were microscopically observed (60X). Representative images were captured using a gel documentation system.^28^

### 2.9 Protease Activity

Protease activity of *C. albicans* were determined using bovine serum albumin (0.2% BSA) agar; sub-MIC concentrations of CEO were added in the BSA agar, and then, 5 μL of *C. albicans* was spotted at the center of the plates and incubated at 37 °C for 72 h. An opaque milky-white ring around the colonies was used as an indicator of protease activity.^29^ Pz was calculated as the ratio of colony diameter to the total diameter of the colony plus the surrounding proteolytic clear zone (Pz = colony diameter / [colony + clear zone diameter]).^30^

Protease activity of *C. albicans* was also assessed using the azocasein assay, in which extracellular proteases hydrolyze the chromogenic substrate azocasein, releasing azo dye-labeled peptides measurable at 440 nm. Cells grown in PDB were transferred to a proteinase-inducing medium containing 0.2% BSA and treated with essential oil at subinhibitory concentrations, along with untreated and solvent controls. Following incubation, cell-free culture supernatants were collected and incubated with azocasein (2%), and the reaction was terminated using TCA before spectrophotometric analysis. Protease activity was normalized to secreted protein content or expressed relative to controls.^31^

### 2.10 GC–MS Analysis of CEO

The essential oils were examined using a Shimadzu gas chromatograph mass spectrometer (QP2020C NX) equipped with a Shimadzu single quadrupole 8030 series mass selective detector and a cross-bond 1,4-bis­(dimethylsiloxy)­phenylene dimethyl polysiloxane SH-Rtx-5MS capillary column (30 m × 0.32 mm, film thickness 0.25 μm). In the split mode (1:5), 1 μL of the diluted essential oil in diethyl ether (1:10 dilution) was injected. The oven temperature ranged from 60 to 250 °C at a rate of 3 °C per minute, while the injector temperature was 240°. The mass detector’s interface temperature was 260 °C, and its ion source temperature was 240 °C. Electron impact ionization (EI) at 70 eV was the mode of ionization. The components were found using an MS Library search (NIIST 17, Wiley 275), mass fragmentation pattern analyses, relative retention indices (RRI), and literature reference (Adams, 2017).

### 2.11 Molecular Docking Study

The 3D conformers of the major phytoconstituents of CEO (cuminaldehyde(33.92%), g-terpinene(14.42%), b-pinene(13.62%), *p*-cymene (12.90%), a-terpinen-7-al (5.77%), 10-*epi*-β-acoradiene­(4.41%), g-terpinen-7-al (2.40%), a- pinene(1.39%), α-phellandrene (1.03%), and myrcene (0.95%)) were obtained in the sdf format from the PubChem database. The 3D crystal structure of *C. albicans* heat shock Protein 90 (Hsp90) bound to radicicol (PDB ID: 6CJL) was downloaded from the RCSB Protein Data Bank (https://www.uniprot.org/). The 3D homology modeled structural coordinates of Sap4 (AF-B8YPH8-F1), Sap5 (AF-P43094-F1), and Sap6 (AF-P43095-F1) proteins of *C. albicans* were retrieved from the AlphaFold database. The protein was prepared by removing water molecules and any bound ligands, followed by the addition of polar hydrogens and Kollman charges using Auto Dock Tools 1.1.2. The major constituents of CEO were obtained from PubChem (https://pubchem.ncbi.nlm.nih.gov/), energy minimized using Chemsketch (https://www.acdlabs.com/), and converted to the pdbqt format using AutoDock Tools. The catalytic aspartyl residues of each Sap within its active site were surrounded by the grid box to define the binding site. Molecular docking was carried out using Auto Dock Vina, and the binding affinity in kcal/mol for each ligand with the target proteins was recorded. The best-scoring conformations were selected based on binding affinity. The docking poses were visualized and analyzed using PyMOL and Discovery Studio Visualizer to study the noncovalent interactions such as hydrogen bonding and hydrophobic contacts between the residues in the binding site of the target protein and the CEO constituents.

### 2.12 Quantification of Biofilm Formation

The effect of the CEO on biofilm formation of *C. albicans* was analyzed by a modified crystal violet assay in PDB.^32^ The *C. albicans* biofilm was developed on sterile 96-well plates by adding cultures (OD 0.2) along with CEO (0.5% to 0.007%) and incubated at 37 °C for 24 h. Wells were washed thrice with PBS to eliminate unattached cells. Biofilms formed on the well were stained with 1% crystal violet for 20 min, washed thrice with distilled water, and dried at room temperature. Acetic acid (33%) was added to each well and incubated for 20 min at room temperature, and the absorbances were measured at 600 nm.

### 2.13 Biofilm Formation in Different Physiological Conditions

To study the effect of CEO on biofilm formation at different physiological conditions, RPMI medium and simulated fluids such as tear fluid,^33^ artificial urine (pH 7),^34^ and artificial saliva^35^ were used.

Artificial urine was prepared using urea (333 mM), sodium chloride (90 mM), potassium chloride (25 mM), ammonium chloride (17 mM), creatinine (10 mM), calcium chloride dihydrate (6.5 mM), magnesium sulfate heptahydrate (3 mM), sodium sulfate (2.5 mM), sodium dihydrogen phosphate (2.8 mM), disodium hydrogen phosphate (4.0 mM), and sodium citrate (1.0 mM) in 800 mL of Milli-Q water, and pH was adjusted to 7 ± 0.1. The solution was filtered using a 0.22 μm membrane filter and stored at 4 °C in amber bottles for up to 7 days.

Artificial tear pH 7.4 and artificial saliva were prepared as described earlier by Rändler et al. (2010). The composition including sodium chloride (10.96 mM), sodium bicarbonate (2.38 mM), calcium chloride dihydrate (0.54 mM), lysozyme (2.0 g/L), urea (1.00 mM), glucose (5.55 mM), lactoferrin (0.9 g/L), and mucin (2.0 g/L), with pH adjusted to 7.4, was filtered through a 0.22 μm membrane filter and stored at 4 °C.

Artificial saliva was prepared by dissolving sodium chloride (125 mM), potassium chloride (20 mM), calcium chloride dihydrate (1.5 mM), magnesium chloride hexahydrate (0.5 mM), potassium dihydrogen phosphate (4.0 mM), sodium bicarbonate (15 mM), urea (2.0 mM), and mucin (0.2% w/v) in Milli-Q water. The pH was adjusted to 6.8 ± 0.1, and the solution was sterilized using a 0.2 μm membrane filter and stored at 4 °C.

### 2.14 Gene Expression Studies

Fungal RNA was extracted after treating *C. albicans* with sub-MIC concentrations of CEO using a fungal extraction kit (Origin, India). Complementary DNA was synthesized using a cDNA Synthesis Kit (Origin India), followed by amplification with 2X Real-Time PCR Master mix in a final volume of 20 μL (Origin India). Genes encoding potential virulence factors in the *C. albicans* biofilm, including agglutinin-like sequences (*ALS3*), hyphal cell wall specific genes (*HWP1*), the morphogenesis pathway regulatory gene (*RAS1*), and the molecular chaperone gene (*HSP90*), were evaluated (Primers prepared by Eurofines Genomics India Pvt. Ltd.). RT-PCR was performed using the QuantStudio 5 RT-PCR system (Applied Biosystems by Thermo Fisher Scientific), under 40 cycles of denaturation and extension, during which fluorescence signals were captured. *18S rRNA* was selected as a housekeeping gene.^36^ The relative fold changes in gene expression levels were calculated using the comparative Ct method (ΔΔ*C*_t_). Primer sequences are shown in Supporting Information Table 1.

### 2.15 ROS Production

ROS production by *C. albicans* was assessed using the ROS-sensitive probe 2,7-dichlorodihydrofluorescein (0.5 μM). *C. albicans* suspension (OD 0.2) was centrifuged at 5000 rpm for 5 min, and pellets were resuspended in PBS. Cells were treated with CEO (0.25% to 0.015%) and incubated at 37 °C for 4 h. DCFH-DA stain (1 μL) was added and incubated at room temperature for 30 min, and fluorescence intensity was measured (485 nm excitation and 530 nm emission).^37^

### 2.16 *C. albicans* and THP-1 Macrophage Co-culture for Germ Tube Formation, Phagocytosis Assay, and Cytokine Analysis

Overnight cultures of *C. albicans* grown in potato dextrose broth were centrifuged at 7000 rpm for 10 min, and pellets were resuspended in PBS, washed twice, and resuspended in serum-free RPMI media, adjusted to an OD of 0.2.

THP-1 cells were maintained in RPMI-1640 medium (Gibco) supplemented with 10% (v/v) fetal bovine serum (FBS; Gibco) at 37 °C in a humidified incubator with 5% CO_2_. For interaction studies, cells were seeded in 96-well plates and differentiated into macrophages as described previously. Prior to infection, differentiated macrophages were washed and maintained in antibiotic-free, PMA-free medium for 24 h.

*C. albicans* was grown overnight in potato dextrose broth, harvested by centrifugation (7000 rpm, 10 min), washed twice with phosphate-buffered saline (PBS), and resuspended in serum-free RPMI-1640 medium. The fungal suspension was adjusted to an OD(600) of 0.2. Macrophages were infected with *C. albicans* at a multiplicity of infection (MOI) of 20:1 (*C. albicans*: macrophage) in the presence or absence of sub-MIC concentrations of cumin essential oil (CEO) and incubated at 37 °C with 5% CO_2_. Germ tube and hyphal formation during macrophage–fungal interaction were monitored microscopically (Olympus TH4-200) at defined time intervals as described previously.

For phagocytosis assays, differentiated THP-1 macrophages were infected with *Candida albicans* ^38^ (MOI 20:1) in the presence of CEO and DMSO control and incubated at 37 °C for 4 h. Non-adhered and noninternalized yeast cells were removed by PBS washing, followed by brief treatment with 0.05% sodium dodecyl sulfate (SDS) for 1 min to lyse the macrophage. Adhered and internalized *C. albicans* cells released from lysed macrophages were plated on potato dextrose agar (PDA). Plates were incubated at 30 °C for 24–48 h, and colony-forming units (CFU) were enumerated.^39^

For the cytokine study, the co-culture experiments were performed as described earlier. Culture supernatants were collected from the co-culture experiment, centrifuged at 7000 rpm for 10 min, and kept at −80 °C until analysis. Cytokine profiling was done for pro-inflammatory (TNF-α, IL-1β, IL-23, IL-12, and IL-6) and anti-inflammatory cytokines (IL-10) by ELISA (Origin, India) as per the manufacturer’s direction.^40^

### 2.17 Statistical Analysis

All data presented in the figures represent the mean of three independent experiments. The data were analyzed using GraphPad Prism software 10.1.2 (GraphPad Software Inc., CA, USA). The results were expressed as the mean ± SD. The statistical significance of the differences between the groups was determined by one-way or two-way ANOVA.

## 3 Result

### 3.1 Antifungal Effect of CEO on Growth of *C. albicans*

To test the antifungal potential of CEO against *C. albicans* (MTCC 183), a well diffusion assay was performed. A distinct zone of inhibition surrounding the CEO well confirmed its antifungal activity, whereas no inhibition was observed with the DMSO control (Figure 1A). The minimum inhibitory concentration (MIC) and minimum fungicidal concentration (MFC) were determined using the broth microdilution method (Table 1). CEO exhibited a clear dose-dependent inhibition of *C. albicans* growth with the tested concentration range (0.03–0.5% v/v). The MIC and MFC were 0.25% (v/v) and 0.5% (v/v), respectively. No significant effect on *C. albicans* growth was observed in the presence of 0.5% DMSO (Figure 1B).

**Figure 1:**
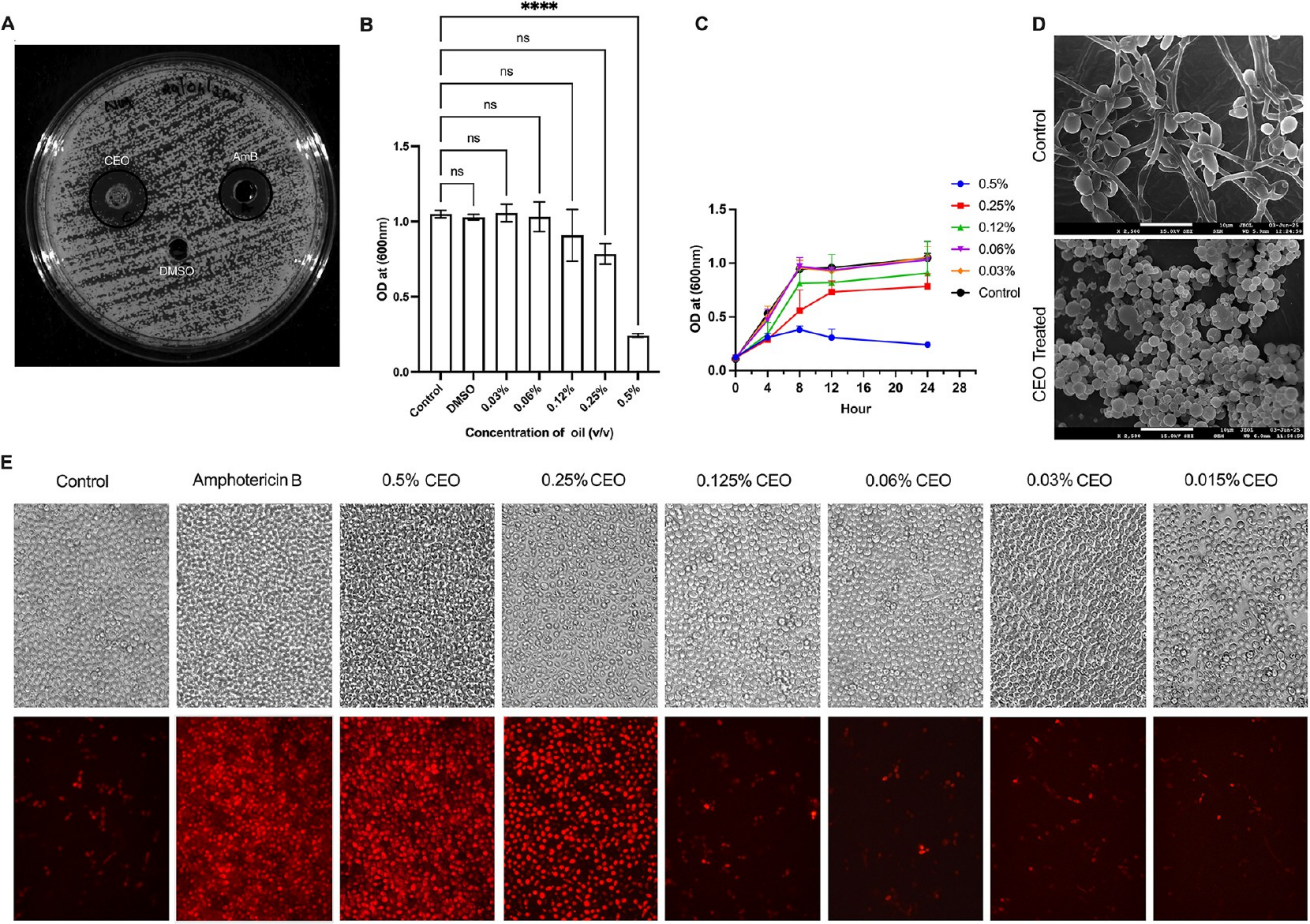
Effect of CBO on growth of *C. albicans*. (A) Agar well diffusion assay, showing the zone of inhibition by CEO, amphotericin B, and DMSO. (B) Broth dilution assay, showing the dose-dependent effect of CEO on *C. albicans.* (C) Growth kinetics of *C. albicans* treated with different concentrations of CBO. (D) SEM analysis of CEO-treated *C. albicans*. (E) Propidium Iodide staining of *C. albicans* treated with different concentrations of CEO. Data represent the mean ± SD of three independent experiments. Statistical significance was determined by one-way ANOVA (*p < 0.05, **p < 0.01, ****p < 0.0001; ns, not significant).

**Table 1:** Minimum Inhibitory and Minimum Fungicidal Effect of CEO

Essential Oils	Scientific Name	MIC %	MFC %
Cumin	Cuminum cyminum	0.25	0.50

### 3.2 Time-Killing Assay

The time-kill assay results demonstrate that 0.5% CEO achieved complete killing of *C. albicans*, indicating potent fungicidal activity. Conversely, none of the lower concentrations tested showed any fungicidal effect at 4, 8, 12, or 24 h. These results highlight the concentration-dependent nature of antifungal activity of CEO, emphasizing its potential as a natural antimicrobial agent (Figure 1C).

SEM analysis revealed that treatment with 0.25% CEO leads to cluster formation of yeast cells, compared to extensive hyphae with yeast cells in the untreated control (Figure 1D).

PI staining demonstrated that 0.25% and 0.5% CEO cause a significant increase in membrane permeabilization, resulting in cellular accumulation of PI. A dose-dependent membrane permeabilization by CEO in *C. albicans* is shown by cellular accumulation of PI (Figure 1E). However, membrane permeabilization and accumulation of PI were significantly reduced when treated with sub-MIC concentrations of CEO (Figure 1E).

### 3.3 Essential Oil Inhibited the Formation of Wrinkled Colony Morphology

Under standard growth conditions, *C. albicans* colonies typically exhibit pronounced wrinkled surface colonies, which facilitate improved access to oxygen, essential for hyphal development and biofilm formation.^41^ When sub-MIC concentration of CEO (0.03% and 0.015%) was incorporated in PDA agar, colonies displayed smooth morphology, indicating a decrease in filamentous growth and suppression of the development of wrinkled colony morphology compared to untreated controls. *C. albicans* colonies displayed a smooth, flattened, and glossy surface morphology with well-defined margins in untreated control. The treated colonies lacked wrinkles surface folds and appeared to be more compact and homogeneous, indicating a significant reduction in filamentous growth. The loss of the wrinkled architecture suggests suppression of hyphal formation compared to untreated controls (Figure 2A).

**Figure 2:**
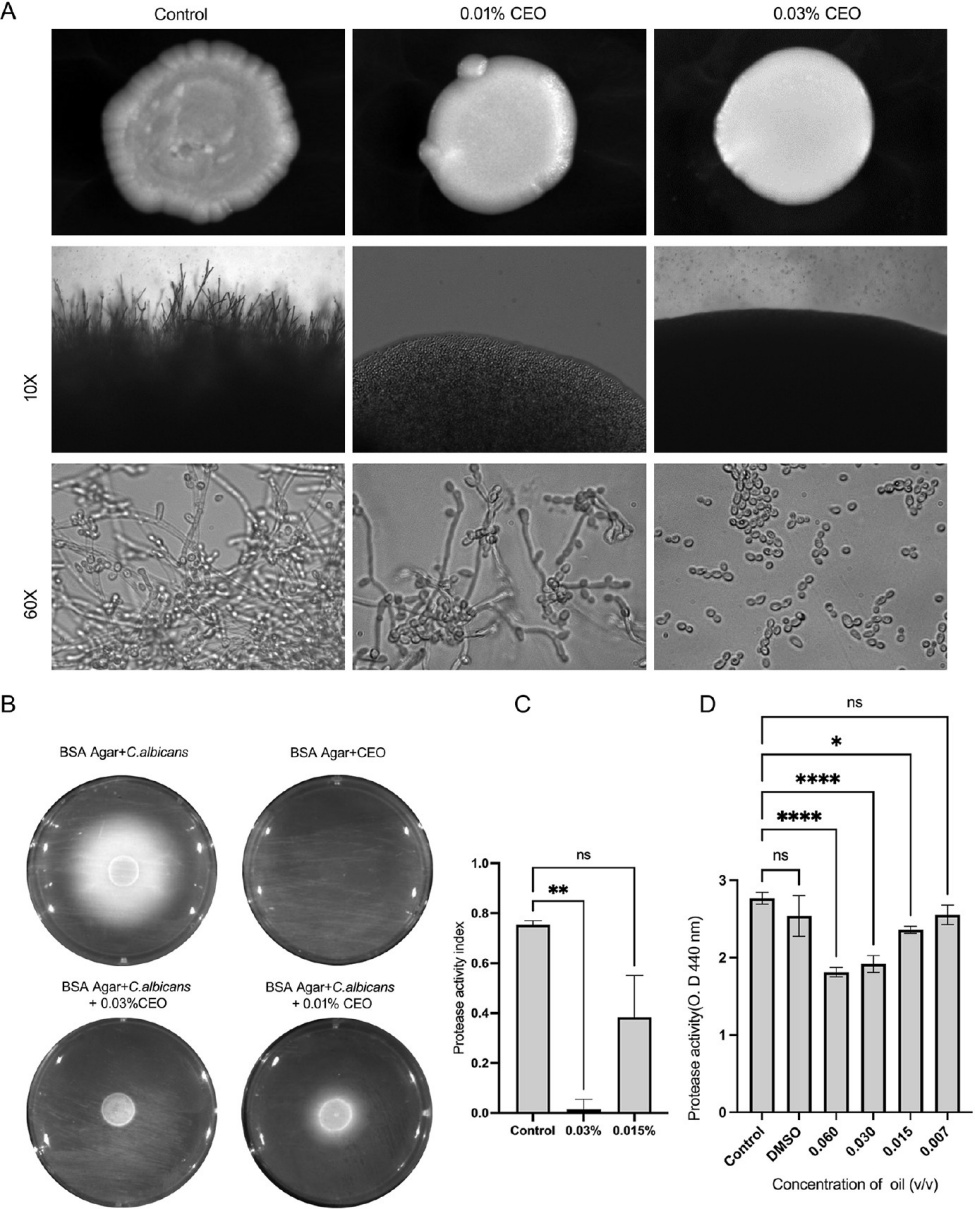
Effect of cumin essential oil (CEO) on wrinkled colony morphology, filamentation, and extracellular protease activity of *C. albicans*. (A) Representative images of *C. albicans* colonies grown in the presence of sub-MIC concentrations of CEO (0.01% and 0.03%). as observed at 10×, and the wet mount image of the colony at 60× magnification. (B) Extracellular protease activity on BSA agar, visualized by opaque precipitation zones surrounding colonies, which were markedly reduced following CEO treatment. (C) Quantification of protease activity index showing significant inhibition at sub-MIC concentrations of CEO. (D) Azocasein-based protease activity assay measuring extracellular protease activity with different concentrations of CEO. Data represent the mean ± SD of three independent experiments. Statistical significance was determined by one-way ANOVA (*p < 0.05, **p < 0.01, ****p < 0.0001; ns, not significant).

### 3.4 CEO Reduced the Protease Activity of *C. albicans*

In the bovine serum albumin (BSA) agar plate assay, *C. albicans* demonstrated extracellular protease activity, indicated by the formation of a distinctive white, opaque, milky ring around colonies. When *C. albicans* was inoculated onto BSA agar supplemented with sub-MIC doses of CEO (0.03% and 0.015%), there was a significant reduction in protease activity, indicated by a reduction in the diameter of the white precipitation ring compared to the untreated control. This dose-dependent suppression was further supported by quantitative protease activity index analysis, which revealed a significant decrease in protease activity, with 94.3% inhibition at 0.03% CEO and 49% inhibition at 0.015% compared to the untreated control. Incorporation of CEO alone did not produce any precipitation or clearance zones on uninoculated BSA agar plates, indicating that the observed reduction in protease activity is unlikely due to direct BSA–oil interaction (Figure 2B,C).

Extracellular protease activity of *C. albicans* was also assessed using an azocasein-based assay to evaluate the effect of sub-MIC concentrations of CEO. Treatment with 0.03% and 0.15% CEO exhibited 30.7% and 14.7% reductions in protease activity compared to the untreated and DMSO control, respectively. These results demonstrate that CEO effectively suppresses extracellular protease secretion in *C. albicans* (Figure 2D).

### 3.5 CEO Reduced Yeast-to-Hyphal Transition of *C. albicans*

Under hyphal-inducing conditions (supplemented with 20% FBS), the effect of sub-MIC concentrations of CEO on the yeast-to-hyphal transition of *C. albicans* was microscopically observed. A highly entangled hyphal network with intruded yeast cell aggregates was observed in the untreated control, whereas dispersed yeast cells without germ tube formation and hyphae were observed in sub-MIC concentrations of CEO, suggesting substantial interference of CEO with key virulence mechanisms of the pathogen. The effect of CEO on the morphological transition shown in Figure 3A,B. Untreated and DMSO-treated *C. albicans* exhibited comparable levels of germ tube formation (65%). In contrast, CEO treatment led to a significant reduction in germ tube formation, with 0.03% and 0.01% CEO resulting in germ tube formation of 0% and ∼4%, respectively. These results demonstrate the strong antimorphogenetic potential of CEO against *C. albicans*.

**Figure 3:**
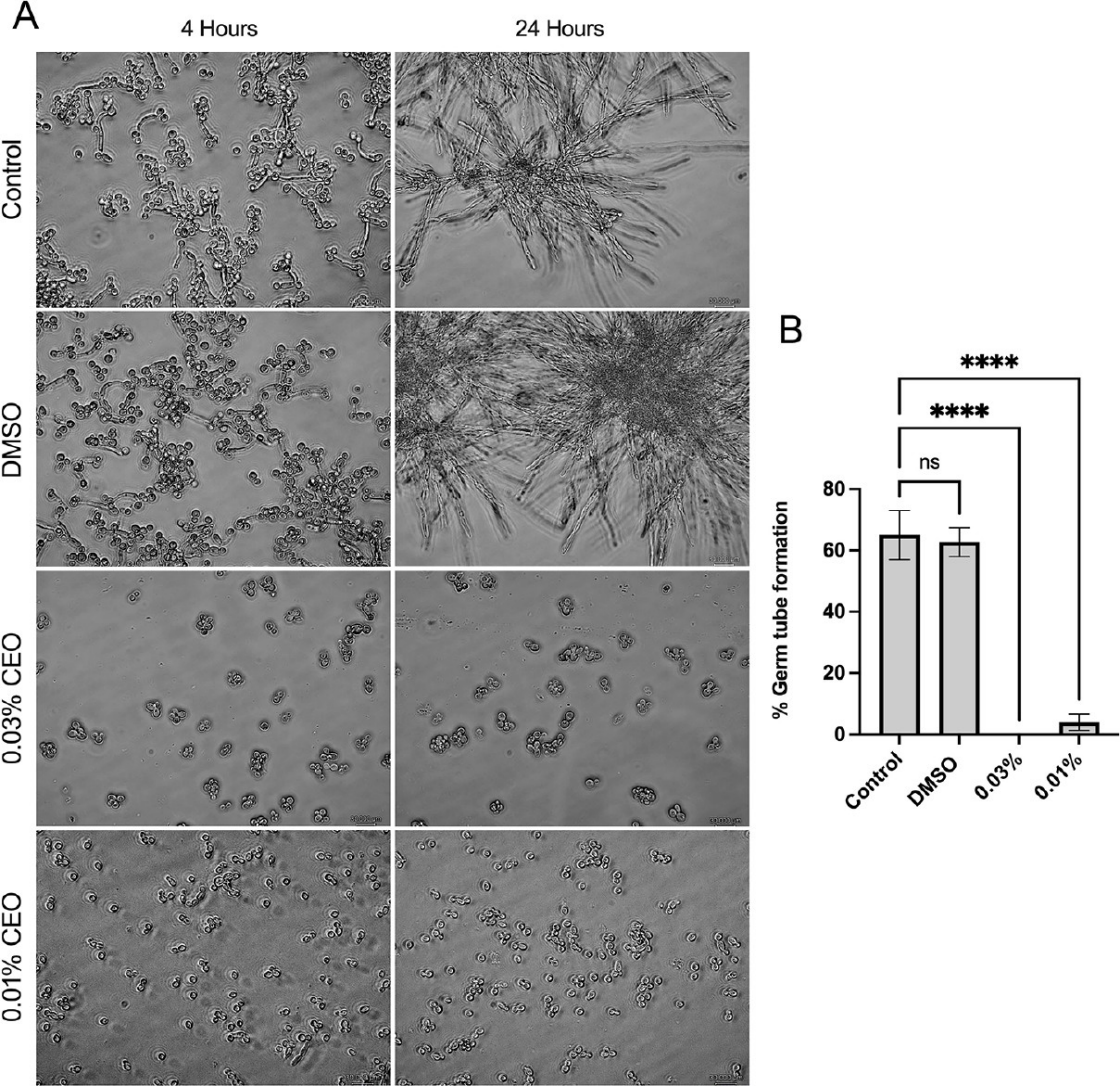
Effect of CEO on the yeast-to-hyphal transition in *C. albicans*. (A). Representative phase-contrast microscopic images (40× magnification) showing morphological changes at 4 and 24 h in untreated control, DMSO control, and CEO treatment conditions. Untreated and DMSO-treated cells exhibited extensive hyphal and filamentous growth over time, whereas treatment with sub-MIC concentrations of CEO (0.03% and 0.01%) resulted in a marked inhibition of germ tube formation and hyphal elongation, with cells predominantly retaining the yeast form. (B). Quantification of germ tube formation of *C. albicans* following CEO treatment. Untreated and DMSO-treated cells showed comparable levels of germ tube formation, whereas CEO treatment resulted in a significant reduction. Data represent the mean ± SD of three independent experiments. Statistical significance was determined using one-way ANOVA (****p < 0.0001; ns, not significant).

### 3.6 GC–MS Analysis

GC–MS analysis of (CEO) revealed a profile with monoterpene and aromatic aldehyde constituents (Figure S1): cuminaldehyde (33.92%), **γ**-terpinene (14.42%), β-pinene (13.62%), *p*-cymene (12.90%), α-terpinen-7-al (5.77%), 10-*epi*-β-acoradiene (4.41%) γ-terpinen-7-al (2.40%), α-pinene (1.39%), α-phellandrene (1.03%), and myrcene (0.95%). Compounds were identified mainly by comparison of mass spectra and RRI values with the reference data reported by Adams and further confirmed using the NIST mass spectral library. Relative amounts were determined by peak area normalization. In line with the previously documented chemical profile, a cuminaldehyde-, terpinene-, pinene-, and cymene -rich chemotype was validated (Table 2, Figure S1).

**Table 2:** GC–MS Analysis of CEO^a^

No	Compound	RT	Area (%)	RI (exp)	RI (lit)
1	Cuminaldehyde	18.104	33.92	1248.14	1238
2	**γ**-Terpinene	10.158	14.42	1062.33	1054
3	β-Pinene	7.180	13.62	978.87	974
4	*p*-Cymene	8.856	12.90	1028.20	1020
5	α-Terpinen-7-al	19.711	5.77	1284.07	1283
6	10-*epi*-β-Acoradiene	27.605	4.41	1470.73	1474
7	γ-Terpinen-7-al	19.956	2.40	1288.06	1290
8	α-Pinene	5.811	1.39	930.66	932
9	α-Phellandrene	8.009	1.03	1006.02	1002
10	Myrcene	7.542	0.95	991.6	988

a Table lists the identified compounds along with their retention times (RT), experimental retention indices (RI (exp)), and literature retention indices (RI (lit)).

### 3.7 *In silico* Docking Studies of Hsp90 with the Main Constituents of CEO

Hsp90 has recently been identified as a key molecular chaperone in eukaryotes and plays a crucial role in fungal drug resistance by acting as a thermal regulator that controls the morphological transition of *C. albicans*, contributing to biofilm-mediated resistance.^6^ Fungal Hsp90 inhibitors with low toxicity to human cells still remain limited. Thus, exploring novel antivirulent agents that can inhibit Hsp90 may represents a potential therapeutic strategy to combat candidiasis. To further corroborate the literature-derived evidence, a preliminary computational study was performed to predict the possible binding potential of CEO to Hsp90 by using molecular docking studies.

Major components of CEO were docked into the active site of Hsp90 of *C. albicans*. Docking poses revealed the interaction of compounds with the nucleotide-binding domain (NBD) of *C. albicans* Hsp90. The 2D interaction diagram showed multiple noncovalent interactions between CEO components and Hsp90 NBD domain residues (Figure 4). Compounds 10-*epi*-β-acoradiene (−6.036 kcal/mol), α-phellandrene (−5.907 kcal/mol), *p*-cymene (−5.826 kcal/mol), γ-terpinene (−5.765 kcal/mol), and cuminaldehyde (−5.707 kcal/mol) have slightly higher binding potential than the other CEO compounds (Table 3 and Supporting Information Table 2). The compounds being structurally small, they exhibited lower binding affinities; however, they established hydrogen bonds and van der Waals interactions with different amino acid residues in the NBD of Hsp90, which involved the conventional hydrogen bond, carbon hydrogen bond, alkyl, Pi-alkyl, Pi-sigma, and Pi–sulfur interactions. The molecular docking studies were validated by redocking radicicol, a known Hsp90 inhibitor, into the NBD domain of Hsp90. Radicicol exhibited very similar conformation with respect to its cocrystallized pose. The CEO constituents were also observed to be bound in the same binding cavity as radicicol and established interactions with residues in the Hsp90 NBD like radicicol. 10-*epi*-β-Acoradiene, having the highest binding affinity with respect to other CEO components, showed interactions with Ala44 and Met87 similar to radicicol. α-Phellandrene, which is another component having the second-best binding affinity, also showed interaction with Met87, as seen in the radicicol-based interaction with Hsp90. These results demonstrated that CEO constituents can possibly interact with Hsp90 of *C. albicans,* reducing the germ tube formation with no hyphae.

**Figure 4:**
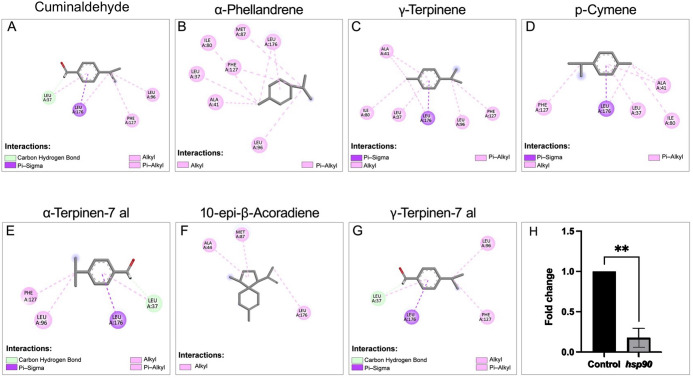
2D interaction maps illustrating the binding modes of major constituents of CEO with *C. albicans* Hsp90 obtained from molecular docking analysis. (A) Cuminaldehyde, (B) α-phellandrene, (C) γ-terpinene, (D) *p*-cymene, (E) α-terpinen-7-al, (F) 10-*epi*-β-acoradiene, and (G) γ-terpinen-7-al. The diagrams depict key intermolecular interactions, including hydrogen bonding, hydrophobic contacts, and π–alkyl interactions, between CEO constituents and amino acid residues within the Hsp90 binding pocket. (H) Relative expression levels of *HSP90* were determined by RT-qPCR and normalized to the housekeeping gene (18S rRNA). Data are presented as fold change relative to the untreated control, which was set to 1.0. Unpaired *t*-test analyses are shown as the mean ± SEM. Statistical significance was assessed using a two-tailed unpaired Student’s *t*-test (*P* < 0.0021).

**Table 3:** Molecular Docking Analysis of CEO Constituents with Hsp90 of *C. albicans* ^a^

Main Constituents	Docking Score(kcal/mol)	Interaction with Amino Acid Residues
Cuminaldehyde	–5.707 kcal/mol	LEU A:37, LEU A:176, PHE A:127, LEU A:96
γ-Terpinene	–5.765 kcal/mol	ALA A:41, ILE A:80, LEU A:37, LEU A:176, LEU A:96, PHE A:127
ρ-Cymene	–5.826 kcal/mol	PHE A:127, LEU A:176, LEU A:37, ALA A:41, ILE A:80
α-Terpinen-7-al	–5.623 kcal/mol	PHE A:127, LEU A:96, LEU A:176, LEU A:37
10-epi-β-Acoradiene	–6.036 kcal/mol	ALA A:44, MET A:87, LEU A:176
γ-Terpinen-7-al	–5.688 kcal/mol	LEU A:37, LEU A:176, PHE A:127, LEU A:96
α- Phellandrene	–5.907 kcal/mol	LEU A:96, ALA A:41, LEU A:37, PHE A:127, ILE A:80, MET A:87, LEU A:176

a Key amino acid residues involved in ligand–protein interactions within the Hsp90 binding site are listed for each docked complex.

### 3.8 Real-Time PCR Analysis of *HSP 90*

Quantitative real-time PCR analysis revealed 5.6-fold downregulation of *HSP90* gene expression in *C. albicans* following treatment with subinhibitory concentrations of (CEO) compared to control. As shown in Figure 4H, CEO-treated cells exhibited a marked reduction in *HSP90* transcript levels compared to untreated control cells, indicative of alterations in the transcriptional activity of the *HSP90* gene mediated by CEO and strengthening the evidence obtained from the docking data. The decrease in the level of *HSP90* expression suggests that CEO interferes with fungal morphogenesis and virulence.

### 3.9 Molecular Docking Analysis of *C. albicans* Saps with CEO Components

The main important hydrolytic extracellular enzymes produced by *C. albicans* are Sap, phospholipase B, and lipases. In aspartyl protease, 10 *SAP* genes are key virulence determinants of *C. albicans*. Expression of Sap4, Sap5, and Sap6 is associated with different virulence traits of *C. albicans*, including hyphal formation, adhesion, and phenotypic switching.^29^ To understand the major inhibitory interaction of CEO on aspartyl protease of *C. albicans*, docking studies of major constituents of CEO, along with Sap4, Sap5, and Sap6, were performed.

Results revealed that all six constituents of CEO exhibited favorable binding affinities toward the Saps with a binding affinity ranging from −7.149 to −4.691 kcal/mol (Table 4, Supporting Information Table 3). 10-*epi*-β-Acoradiene consistently showed strong interactions with Sap4, Sap5, and Sap6, with binding affinities of −7.041, −7.149, and −6.166 kcal/mol, respectively (Table 4). Overall, Sap4 and Sap5 exhibited slightly higher binding affinities for CEO constituents than Sap6, suggesting a differential susceptibility of these enzymes toward target inhibition (Figures 5, 6, and 7). Visualization of ligand–protein interactions indicated that the compounds were oriented favorably within the active site, forming potential hydrogen bonds and hydrophobic interactions with key catalytic residues (Figures S5, S6, and S7).

**Table 4:** Molecular Docking Analysis of CEO Constituents with Sap4, Sap5, and Sap6 of *C. albicans* ^a^

Saps	Main Constituents	Docking Score(kcal/mol)	Interaction with Amino Acid Residues
Sap4	α-Terpinen-7-al	–6.037 kcal/mol	TYR A:160, ILE A:199, ASP A:162, SER A:164, SER A:164, TRP A:127, ARG A:196, ALA A:195
	10-epi-β-Acoradiene	–7.041 kcal/mol	TYR A:160, ILE A:199, ALA A:195, ARG A:196, ILE A:106, ILE A:88
	γ-Terpinen-7-al	–5.954 kcal/mol	TYR A:160, ILE A:199, ASP A:162, SER A:164, TRP A:127, ARG A:196, ALA A:195
Sap5	Cuminaldehyde	–5.912 kcal/mol	TYR A:160, ILE A:199, ASP A:162, SER A:164, ARG A:196, TRP A:127, ALA A:195
	10-epi-β-Acoradiene	–7.149 kcal/mol	ARG A:196, TRP A:127, ILE A:88, ILE A:106, ILE A:199, LEU A:30
	γ-Terpinen-7-al	–5.959 kcal/mol	TYR A:160, ILE A:199, ASP A:162, SER A:164, TRP A:127, ARG A:196, ALA A:195
Sap6	γ-Terpinene	–5.237 kcal/mol	TYR A:357, LYS A:35, LEU A:359, TYR A:360
	10-epi-β-Acoradiene	–5.308 kcal/mol	PRO A:52, HIS A:196, TRP A:127, VAL A:34, ARG A:128, LYS A:35
	ρ-Cymene	–6.166 kcal/mol	TYR A:360, LEU A:359, LYS A:35, TYR A:357

a Key amino acid residues involved in ligand–protein interactions within the Sap binding sites are listed for each docked complex.

**Figure 5:**
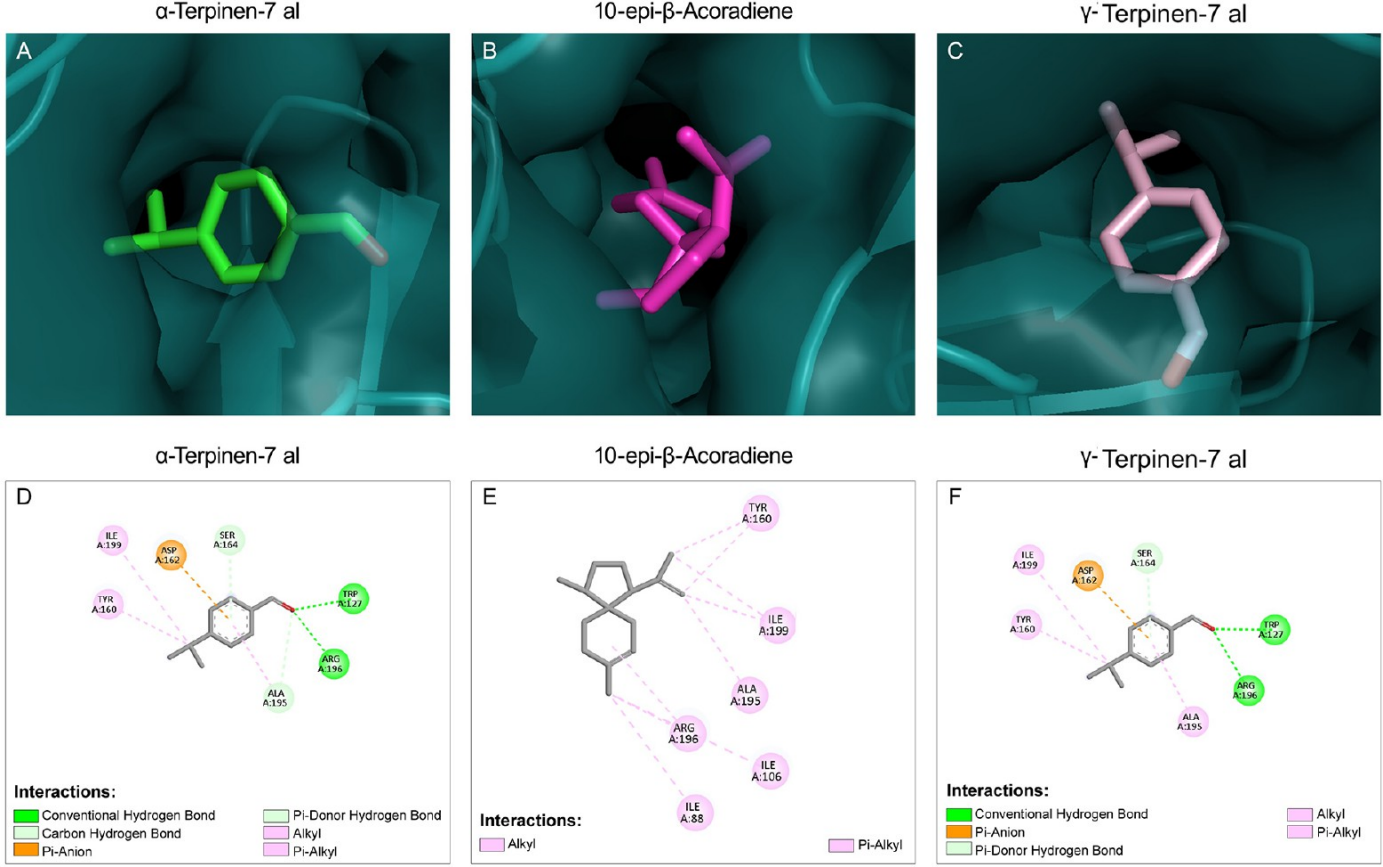
Docked conformation of the best binding components of CEOs with the target proteins. Docking conformation of the top 3 best binding components of CEO with the target protein. (A) α-Terpinen-7-al, with Sap4; (B) 10-*epi*-β- acoradiene with Sap4; and (C) γ-terpinen-7-al with Sap4. The protein surface of Sap4 is shown in cyan, while the CEO components α- terpinen-7-al, 10-*epi*-β-acoradiene, γand -terpinen-7-al are shown in green, magenta, and pink, respectively. (D) 2D structure of α-terpinen-7-al with Sap4; (E) 2D structure of 10-*epi*-β-acoradiene with Sap4; and (F) 2D structure of γ-terpinen-7-al with Sap4.

**Figure 6:**
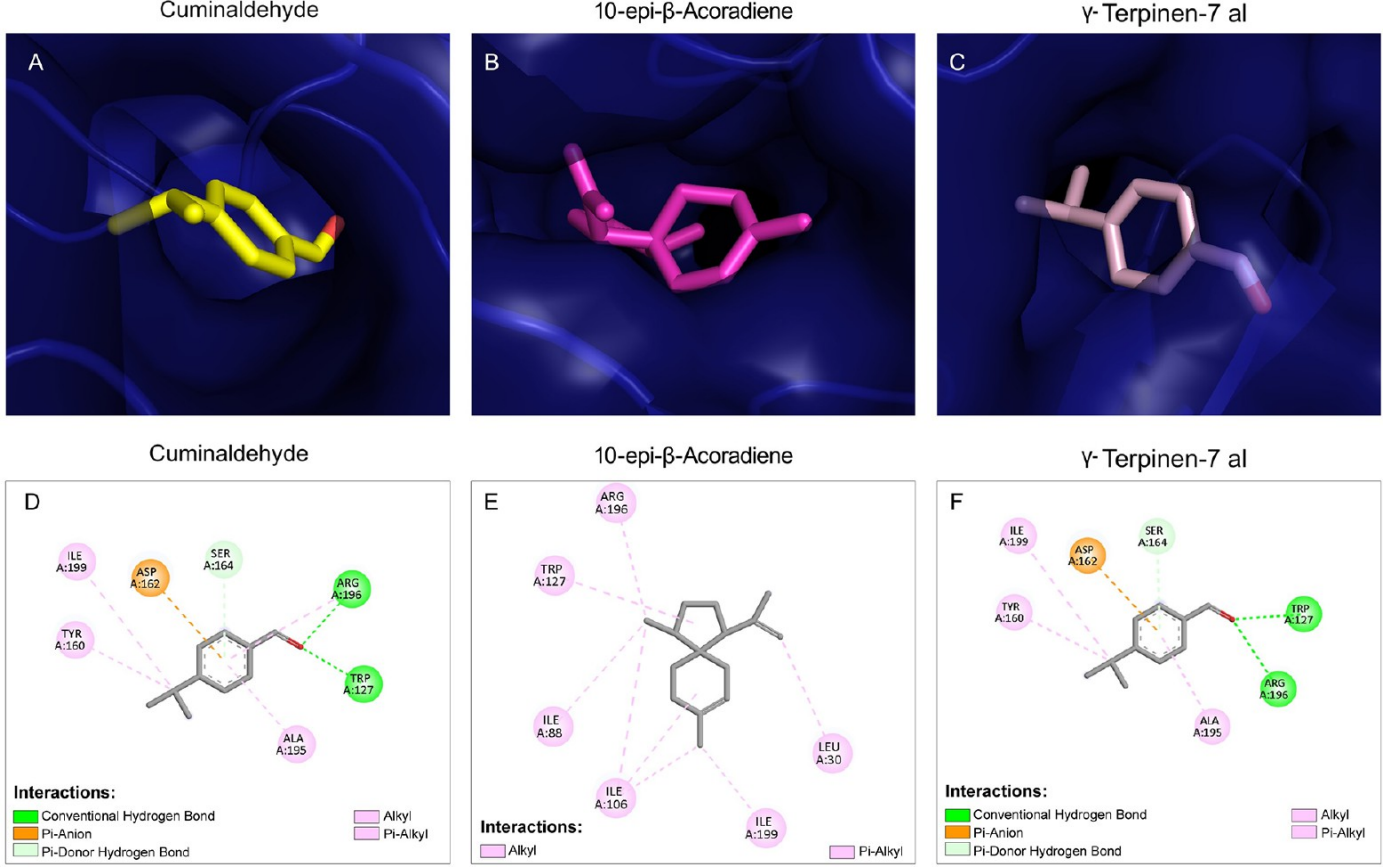
Docked conformation of the top 3 best binding components of CEOs with the target protein. (A) Cuminaldehyde with Sap5; (B) 10-*epi*-β-acoradiene with Sap5; and (C) γ-terpinen-7-al with Sap5. The protein surface of Sap5 is shown in dark blue, while the CEO components cuminaldehyde, 10-*epi*-β-acoradiene, and γ-terpinen-7-al are shown in yellow, magenta, and pink, respectively. (D) 2D structure of cuminaldehyde with Sap5; (E) 2D structure of 10-*epi*-β-acoradiene with Sap5; and (F) 2D structure of γ-terpinen-7-al with Sap5.

**Figure 7:**
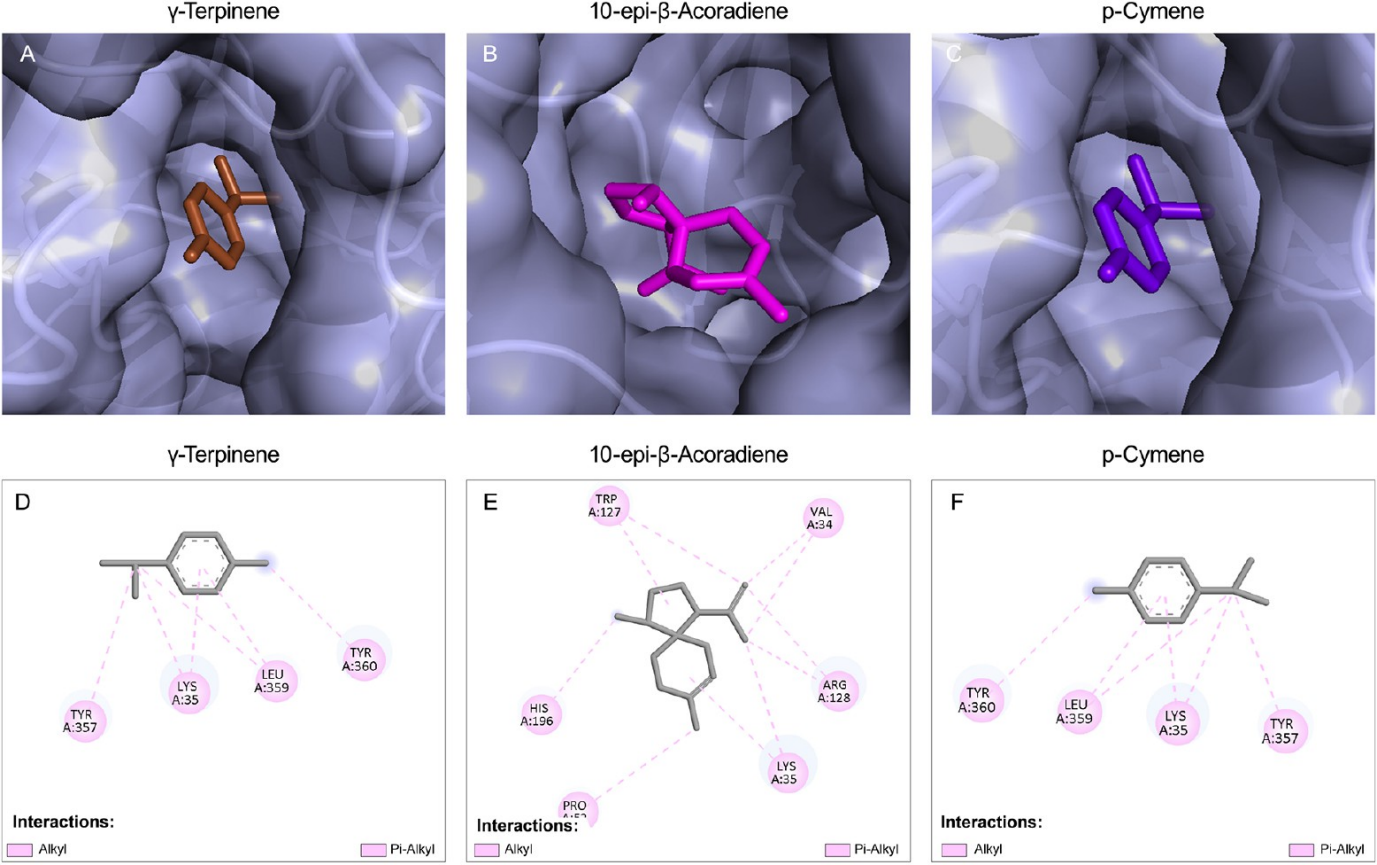
Docked conformation of the top 3 best binding components of CEOs with the target protein. (A) γ-Terpinene with Sap6; (B) 10-*epi*-β acoradiene with Sap6; and (C) *p*-cymene with Sap6. The protein surface of Sap6 is shown in light blue, while the CEO components γ- terpinene, 10-*epi*-β-acoradiene, and *p*-cymene are shown in brown, magenta, and purple blue, respectively. (D) 2D structure of γ-terpinene with Sap6; (E) 2D structure of 10-*epi*-β-acoradiene with Sap6; and (F) 2D structure of *p*-cymene with Sap6.

### 3.10 Effect of CEO on Biofilm Formation of *C. albicans*

CEO significantly inhibited *C. albicans* biofilm formation across two laboratory media and three distinct simulated host environments, demonstrating its broad-spectrum antibiofilm activity. Crystal violet assay and visualizing biofilm development on glass slides consistently showed reduced biofilm in CEO-treated conditions. *C. albicans* grown in PDB formed a thick biofilm in untreated control, in contrast 83% reduction in biofilm upon treatment with CEO. This provided an important baseline for evaluating the antibiofilm capacity of CEO under different simulated conditions (Figure 8A).

**Figure 8:**
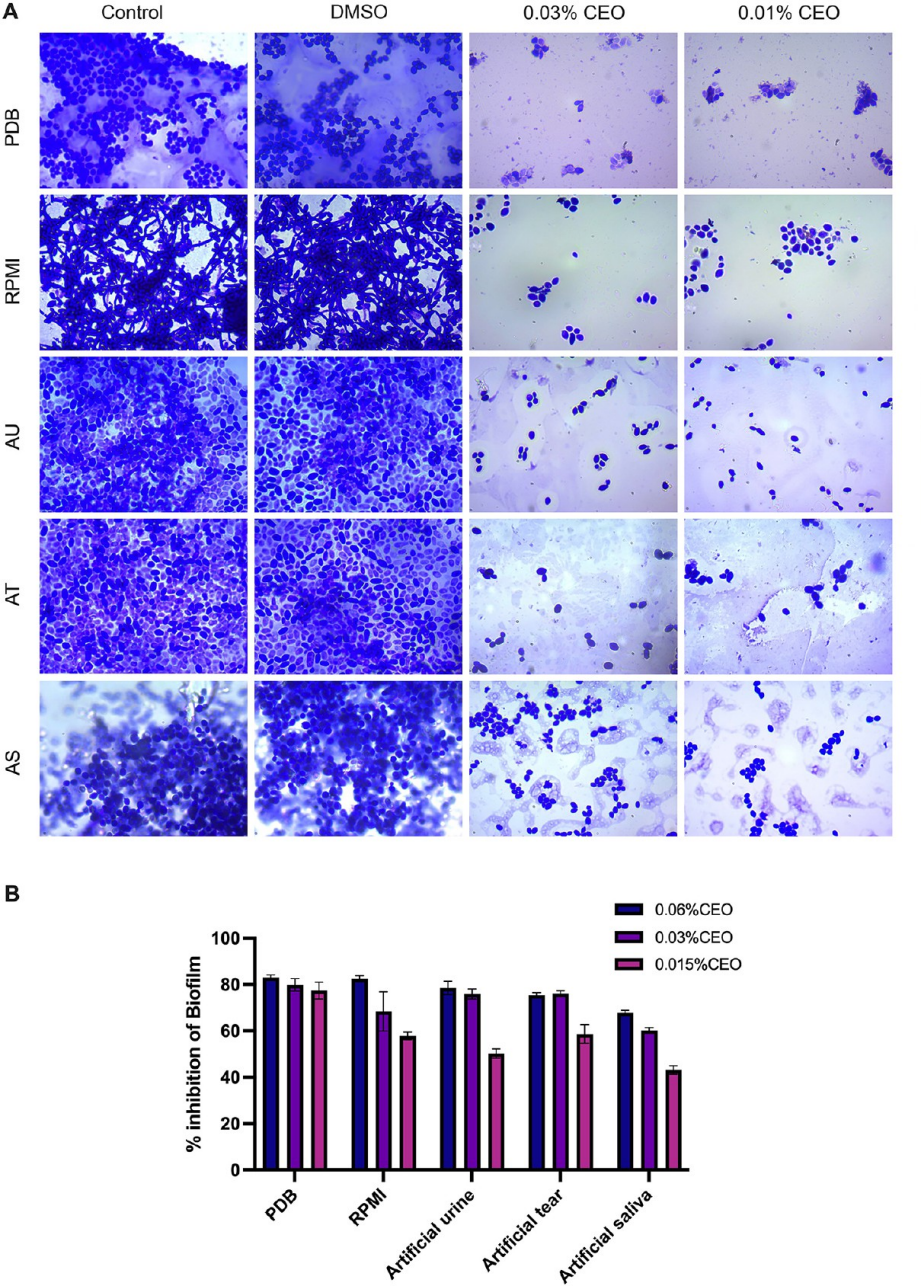
Antibiofilm activity of cumin essential oil (CEO) against *C. albicans*. (A) Representative light microscopic images (40× magnification) showing *C. albicans* biofilms formed under different growth conditions, including potato dextrose broth (PDB), RPMI-1640 medium, artificial urine (AU), artificial tear fluid (AT), and artificial saliva (AS). Biofilm developed in the absence (control) or presence of DMSO and treated with sub-MIC concentrations of CEO (0.03% and 0.01%). Untreated and DMSO-treated samples exhibited a dense, well-organized biofilm with extensive hyphal networks, whereas CEO treatment markedly reduced biofilm biomass and disrupted the biofilm architecture, resulting in sparse and predominantly yeast-form cells. (B) Quantitative analysis of biofilm inhibition expressed as percentage reduction relative to untreated controls. Data represent mean ± SD of three independent experiments.

Furthermore, in RPMI medium, which strongly induces hyphal growth of *C. albicans*, in untreated controls, the biofilm exhibited noticeable yeast and extensive hyphae. Conversely, CEO treatment led to 76% inhibition of biofilm formation, specifically affecting fungal attachment and the yeast-to-hyphae transition required for biofilm development and maturation. This reduction was visually evident in the biofilm formed on glass slides, showing significantly fewer attached cells and minimal hyphal structures (Figure 8A).

The study was further extended to simulated body fluids to assess the biofilm inhibitory potential of CEO for specific clinical applications. In artificial tear fluid, critical for ocular candidiasis and infections related to contact lenses, where biofilm is a significant problem, CEO (0.03%) demonstrated a 76% inhibition of biofilm formation. Microscopic examination of the biofilm formed on glass slides showed significantly reduced fungal adherence. This highlights the potential application of CEO in ophthalmic solutions and contact lens disinfectants (Figure 8A,B).

In simulated urine, this is relevant to urinary catheter-associated infections (CAUTIs) and bladder candidiasis. CEO (0.06%) effectively reduced biofilm formation by 78%. Visualization of the biofilm formed on glass slides correlated with results obtained with the CV assay in CEO-treated urine conditions.^42^

In simulated saliva, relevant to oral candidiasis, common in immunocompromised individuals and denture wearers, biofilms on oral mucosa or prostheses are challenging to treat.^43^ CEO (0.06%) treatment achieved 67% inhibition of biofilm formation in simulated saliva. Microscopic examination of the biofilm formed on glass slides in simulated saliva confirmed that CEO considerably reduced the biofilm (Figure 8A,B).

This wide-ranging evaluation quantified the capacity of CEO to inhibit biofilm formation under a diverse set of conditions and highlighted its broad-spectrum clinical application potential as an antibiofilm agent.

### 3.11 Effect of CEO on ROS Generation in *C. albicans*

To evaluate the reactive oxygen species (ROS) levels during treatment with different concentrations of CEO, *C. albicans* was incubated with the fluorescent probe DCFH-DA, which is sensitive to intracellular redox changes and penetrates the cell membrane. Mitochondrial disruption leads to elevated ROS production, leading to excessive accumulation of ROS, which in turn damages *C. albicans* cells.^44^

As illustrated in Figure 9A,B, treatment of *C. albicans* cells with a higher concentration of CEO (0.25%) resulted in a 6.1-fold increase in fluorescence intensity, indicating a substantial increase in ROS generation compared to that of the untreated control. The CEO disrupts mitochondrial function, which impacts the production of ROS, contributing to fungal cell damage and death. At sub-MIC concentrations of CEO, this disruption is less noticeable, resulting in a corresponding decrease in ROS generation.

**Figure 9:**
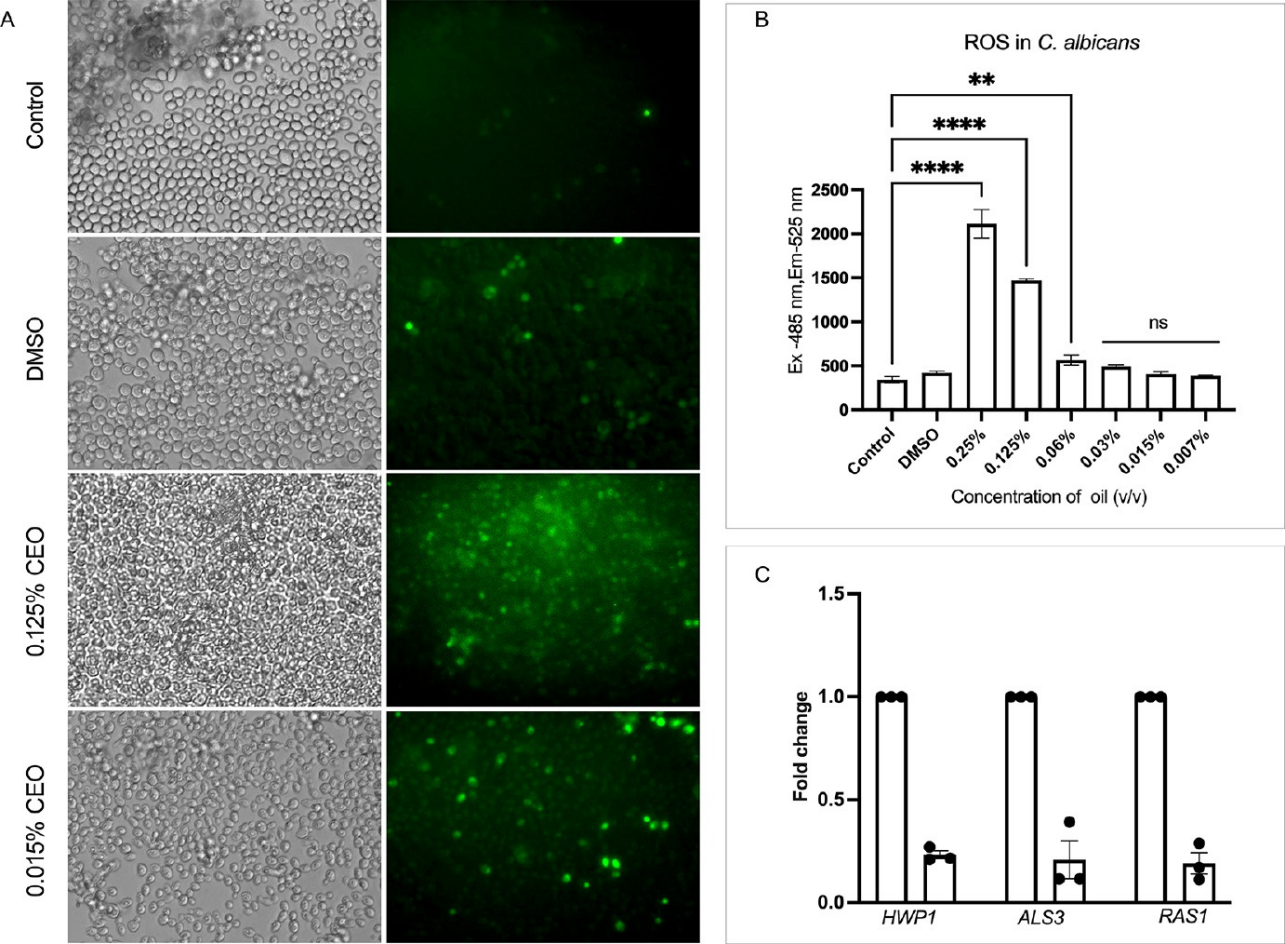
Effect of cumin essential oil (CEO) on reactive oxygen species (ROS) generation and virulence-associated gene expression in *C. albicans*. (A) Representative bright-field and fluorescence microscopic images showing intracellular ROS production in *C. albicans* cells stained with DCFDA following treatment with CEO (0.125% and 0.015%) compared with untreated and DMSO controls. Green fluorescence indicates ROS accumulation. CEO-treated cells exhibited a marked increase in fluorescence intensity, indicating elevated intracellular ROS levels relative to controls. (B) Quantitative analysis of DCF fluorescence intensity is expressed as the mean ± SD of three independent experiments. (C) Relative expression levels of selected virulence- and stress-response-associated genes in *C. albicans* following CEO treatment, as determined by quantitative real-time PCR. Gene expression was normalized to the housekeeping gene. Data represent mean ± SD. Statistical significance was determined using one-way ANOVA (*p* < 0.05, *p* < 0.01, *p* < 0.001).

### 3.12 Effect of CEO on Expression of Genes Involved in Virulence of *C. albicans*

To understand the antivirulence mechanisms of CEO, quantitative real-time PCR (qRT-PCR) was employed to assess the expression of key *C. albicans* virulence genes. Treatment with sub-MIC concentrations of CEO (0.015%) downregulated the expression of hypha-specific genes, such as *ALS3* and *HWP1*. Furthermore, CEO also downregulated the expression of *RAS1*, a major transcription factor in the MAPK pathway known to have a key role in the morphological switch and biofilm formation. Gene expression levels were normalized against the housekeeping gene *18S rRNA* (Figure 9C).

### 3.13 Cytotoxic Effect of CEO

The cytotoxicity of CEO was evaluated by MTT assay using THP-1-derived macrophages and 3T3-L1 and L-929 fibroblast cells. All three cells, treated with CEO (0.25% to 0.007% v/v) for 24 h, demonstrated a dose-dependent effect on cell viability. In THP-1 cells, a significant reduction in cell viability was observed at concentrations ranging from 0.25% to 0.03% compared with untreated controls. In contrast, 0.015% and 0.007% did not significantly affect the viability of the THP-1 macrophages. In mouse L-929 and 3T3-L1 fibroblast cells, CEO at concentrations from 0.06% to 0.007% did not exhibit any cytotoxicity compared to untreated controls (Figure 10A). Accordingly, to safeguard cellular integrity and maintain consistency, 0.015% CEO was chosen for subsequent experiments.

**Figure 10:**
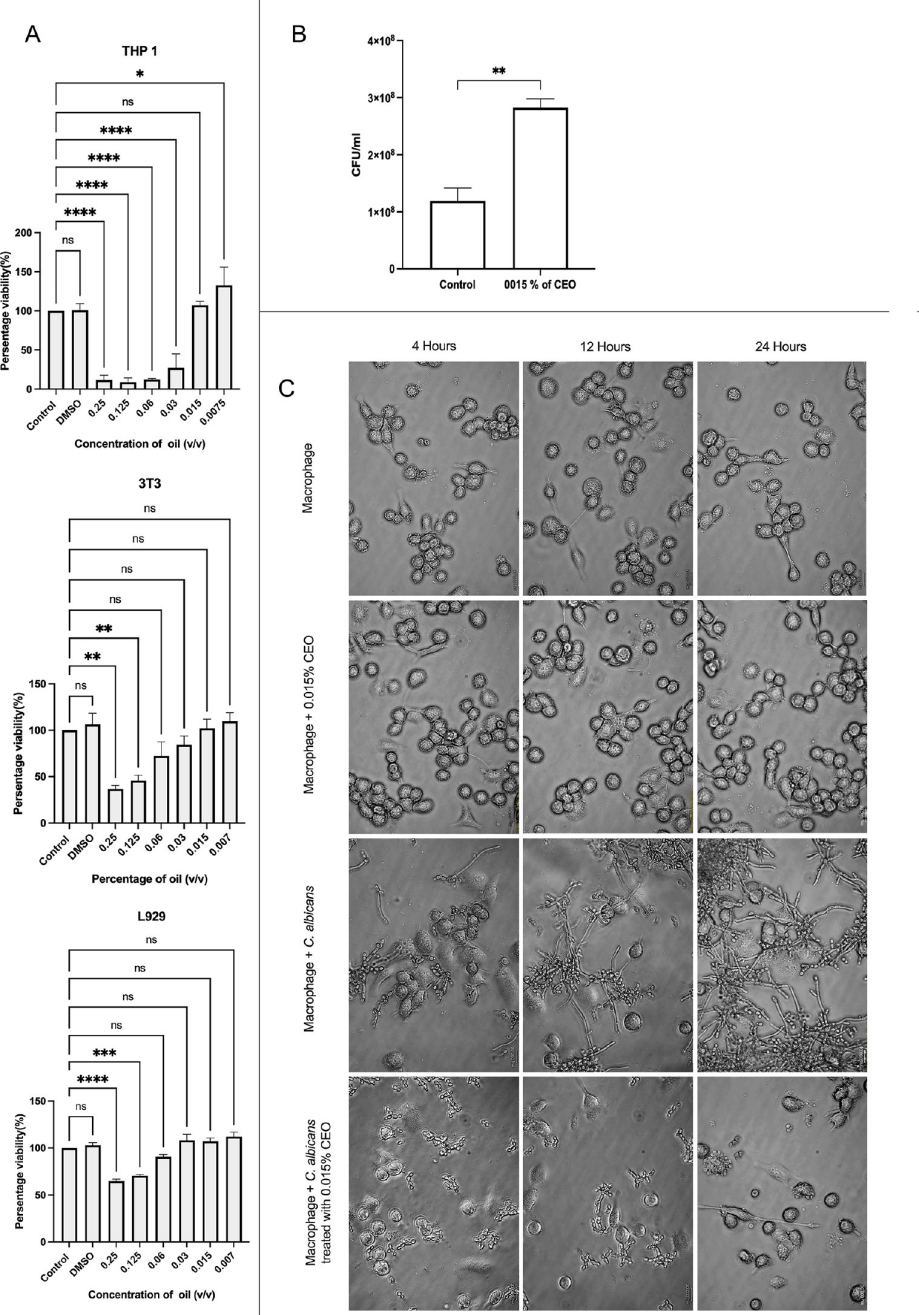
Effect of CEO on host cell viability, *C. albicans* morphogenesis, and phagocytosis. (A) Cytotoxicity of CEO in three mammalian cell lines following treatment with increasing concentrations of CEO. Cell viability was assessed by MTT assay and expressed as a percentage relative to untreated controls. Data represent the mean ± SD from three independent experiments. Statistical significance was determined using one-way ANOVA with Tukey’s multiple comparison test (*p* < 0.05, *p* < 0.01, *p* < 0.001, *p* < 0.0001; ns, not significant). (B) Adherence and phagocytosis of *C. albicans* by THP-1-derived macrophages following treatment with CEO. Phagocytic activity was quantified and expressed as a percentage relative to untreated control. Data represent the mean ± SD from three independent experiments. Statistical significance was determined using Student’s *t*-test (*p* < 0.01). (C) Representative phase-contrast microscopy images showing the effect of CEO (0.015%) on *C. albicans* morphogenesis during macrophage interaction at 4, 12, and 24 h. Untreated cells exhibited extensive hyphal formation, whereas CEO treatment markedly reduced hyphal development.

### 3.14 CEO Alters Phagocytic Response to *C. albicans* in THP1 Macrophages

To investigate the immunomodulatory effects of CEO, we evaluated its influence on macrophage interaction with *C. albicans* using a sub-MIC concentration of CEO. Differentiated THP-1 macrophages were infected with *C. albicans*, and after 1 h of infection, macrophage-associated fungal cells were quantified by lysing the macrophages and enumerating colony-forming units (CFUs/mL). This approach reflects the overall association of *C. albicans* with macrophages, including adhered and internalized cells by phagocytosis. Our results showed that CEO treatment significantly increased the macrophage-associated fungal uptake compared with the untreated control. Treatment with 0.01% CEO resulted in an approximately 3.3-fold increase in macrophage-associated adhered and internalized *C. albicans* compared to the untreated control. These findings suggest that CEO enhances the interaction between macrophages and *C. albicans*, which may facilitate improved host defense against fungal infection (Figure 10B).

### 3.15 Effect of Essential Oil on *C. albicans* Germ Tube Formation in Macrophages

*C. albicans* escapes intracellular killing inside macrophages by transforming from yeast to a filamentous form. This morphological shift of the fungus enables it to rupture the macrophage, which helps in further dissemination into host tissues.^45^ Germ tube formation was assessed by co-culturing macrophages with *C. albicans* treated with CEO (0.015%). At 4 h postincubation, untreated *C. albicans* initiated germ tube formation inside macrophages, which was absent in CEO-treated *C. albicans*. By 12 and 24 h, untreated *C. albicans* exhibited germ tube formation and hyphal extension, leading to complete damage of the macrophage cells. However, in the presence of the CEO, germ tube formation was completely inhibited with minimal damage to the macrophages at 12 and 24 h (Figure 10C).

### 3.16 Essential Oils Modulate Cytokine Production in *C. albicans*–Macrophage Co-cultures

THP-1-derived macrophages infected with *C. albicans* exhibited significantly higher levels of proinflammatory cytokines IL-6 (1.67-fold), TNFα (4.01-fold), IL-1β (3.4-fold), IL-12 (2.8-fold), and IL-23 (6.5-fold) as compared to uninfected controls, indicating that *C. albicans* triggers a robust inflammatory response. When treated with 0.015% CEO, there is a significant suppression of IL-6 (0.5-fold), TNFα (2.1-fold), IL-1β (1.9-fold), IL-12 (1.0-fold), and IL-23 (1.4 fold) compared to untreated *C. albicans* *-*infected macrophages (Figure 11). However, the anti-inflammatory cytokine IL-10 levels were significantly enhanced upon treatment with 0.015% CEO (5.7-fold) compared to untreated *C. albicans* *-*infected macrophages. These results suggest that a reduction in the pro-inflammatory cytokines and increase in anti-inflammatory cytokines are most likely due to the immunomodulatory effect of CEO, which will control the enhanced inflammatory response induced by *C. albicans*. These findings suggest that the CEO not only suppresses *C. albicans*-induced hyperinflammation but not compromising macrophage viability, highlighting its potential as an adjunct antifungal therapy.

**Figure 11:**
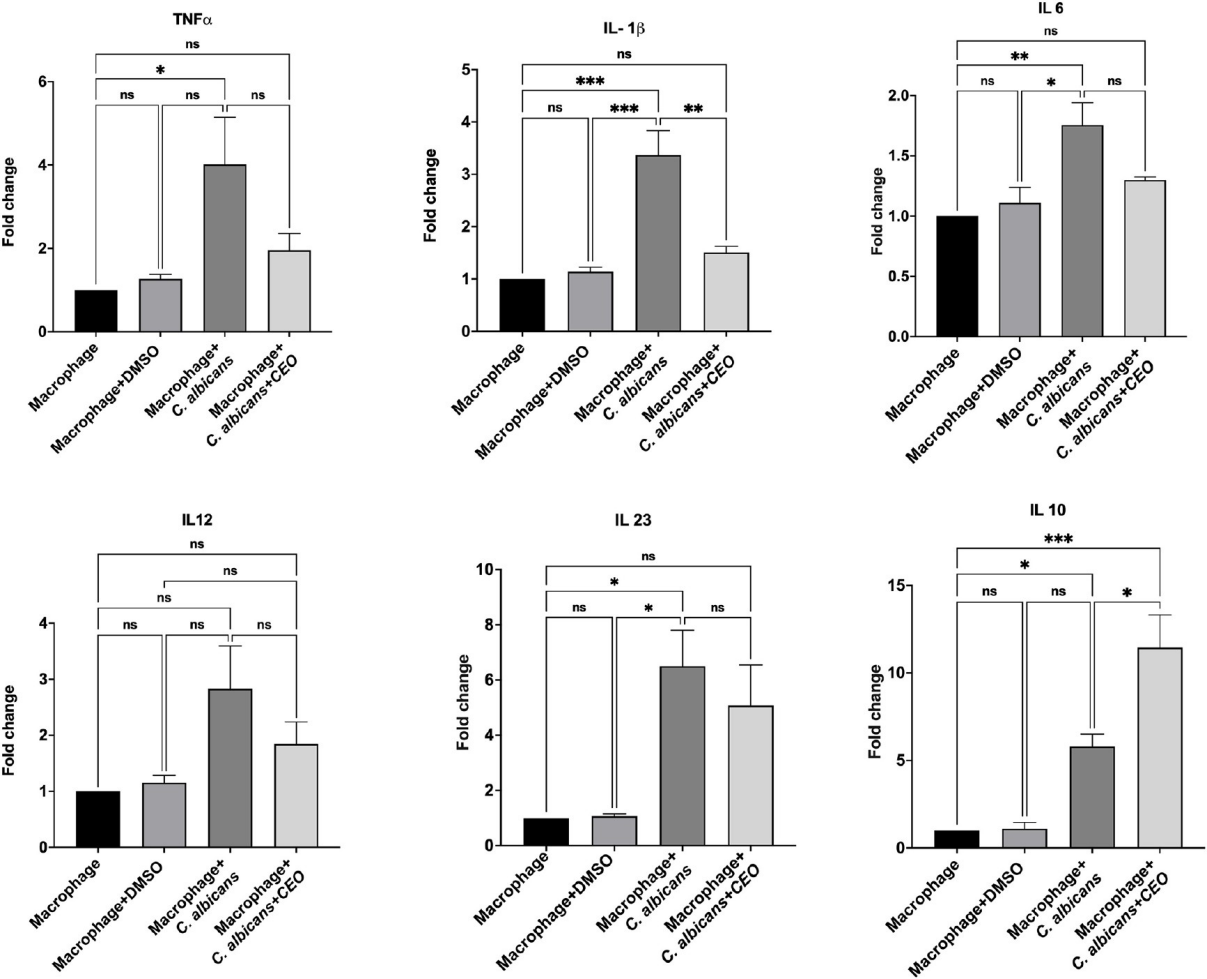
Effect of cumin essential oil (CEO) on cytokine secretion by THP-1-derived macrophages during interaction with *Candida albicans*. Levels of pro-inflammatory cytokines IL-23, TNF-α, IL-6, IL-1β, and IL-4 and anti-inflammatory cytokines IL-10 were quantified in culture supernatants of differentiated THP-1 macrophages under the indicated conditions: macrophages alone, DMSO control, macrophages infected with *C. albicans*, and macrophages infected with *C. albicans* and treated with CEO. Cytokine concentrations were measured using ELISA and expressed as the mean ± SD from three independent experiments. CEO treatment significantly modulated cytokine production in *C. albicans*-stimulated macrophages, indicating an effect on host immune response during fungal challenge. Statistical significance was determined using one-way ANOVA, with *p* < 0.05, *p* < 0.01, *p* < 0.001, and ns indicating not significant.

## 4 Discussion

Antivirulence strategies are increasingly recognized as promising approaches to reduce the strong selective pressure imposed by fungicidal drugs, thereby lowering the likelihood of resistance development in *C. albicans*. By targeting key virulence-associated traits such as yeast-to-hyphal transition, protease production, and biofilm formation, the pathogenic potential of the organism can be attenuated, enabling more effective clearance by the host immune system and contributing to delaying the emergence of drug resistance.^46^ Although the antibacterial and antifungal activities of CEO have been reported previously,^11,15,47^ its antivirulence potential has not been extensively explored. In this context, the present findings demonstrate that CEO exhibits antivirulence activity against *C. albicans* at sub-MIC concentrations. The chemical composition of CEO may contribute to its observed antivirulence activity.

GC–MS analysis confirmed CEO as a cuminaldehyde-rich chemotype, consistent with reported compositions of *C. cyminum* essential oil (cuminaldehyde, *p*-cymene, γ-terpinene, and β-pinene) in the literature.^14,48^ Cuminaldehyde and monoterpene hydrocarbons such as γ-terpinene and *p*-cymene have been associated with antifungal properties, including activity against *Candida* species. The chemically diverse profile of CEO supports a multitarget antivirulence potential, aligning with studies showing essential oil compounds can attenuate fungal pathogenic traits *in vitro*.^48^

Biofilm formation represents an important pathogenic aspect of *C. albicans*, which significantly enhances infection outcome in conditions such as oral thrush,^49^ ocular candidiasis, bloodstream infections, vulvovaginitis, and urinary tract infections.^50^ In the present study, CEO exhibited pronounced antibiofilm activity under different biofilm-inducing conditions, including simulated body fluid environments. These findings are consistent with previous reports describing the antibiofilm properties of essential oils against opportunistic pathogens such as *C. albicans* and *Pseudomonas aeruginosa*.^12,51,52^

The initial important steps in biofilm formation are adherence and germ tube formation. Yeast cells attach to a surface through members of the Als family proteins such as Als3 and Hwp-1. Upon treatment with CEO, gene expression studies using sub-MIC concentrations of CEO demonstrated that genes *ALS3* and *HWP1* are downregulated, indicating that CEO interferes with the initial stages of biofilm formation itself.^53^

The next critical step in *C. albicans* biofilm development following initial adherence is germ tube and hyphal formation. Hyphal growth facilitates tissue penetration and nutrient acquisition by the pathogen. In mature biofilms, the hyphal form becomes the dominant morphological state over yeast cells. One of the most notable effects of sub-MIC-concentration CEO treatment observed in this study was the reduction of the yeast-to-hyphae transition. By inhibiting germ tube formation, CEO disrupts the structural prerequisites for biofilm maturation. Given the central role of filamentation in tissue invasion and immune evasion, its inhibition by CEO likely contributes substantially to the observed reduction in the pathogenic potential. Consistent with this observation, earlier studies have demonstrated that agents capable of suppressing germ tube formation significantly impair biofilm development and virulence in *C. albicans*.^54^

Hsp90, a molecular chaperone in *C. albicans*, acted as a regulator for yeast-to-hyphal transition, in turn leading to biofilm-mediated drug resistance.^55^ The effect of CEO on germ tube formation and biofilm development in *C. albicans* was further investigated in relation to the Hsp90 regulatory pathway. Quantitative real-time PCR analysis revealed a significant downregulation of *HSP90* gene expression in CEO-treated cells compared with the untreated control. In addition, *in silico* docking analysis showed that major constituents of CEO exhibited favorable binding interactions within the binding pocket of Hsp90, overlapping with the region reported for the known Hsp90 inhibitor radicicol.^56^ Although docking results provide only preliminary insights into potential molecular interactions, the observed reduction in *HSP90* transcript levels suggests that CEO may influence Hsp90-associated regulatory pathways involved in morphogenesis and biofilm formation. This observation is consistent with earlier reports showing the downregulation of *HSP*90 gene expression, followed by a subsequent reduction in the virulence of *C. albicans* after treatment with ethnobotanically synthesized gold nanoparticles.^57^

Secreted aspartyl proteases (Saps) are another crucial virulence factor of *C. albicans*, closely linked to hyphal development and biofilm formation.^29^ In the present study, CEO treatment resulted in a reduction in protease activity of *C. albicans*, suggesting a potential effect on protease-associated virulence modulation. To further explore possible molecular interactions, *in silico* docking analysis was performed, which indicated that CEO constituents such as 10-*epi*-β-acoradiene and α-phellandrene exhibited favorable binding interactions with Sap4 and Sap5 through multiple noncovalent interactions. These preliminary observations of computational analysis suggest possible interactions between CEO constituents and Sap proteins. Further experimental studies, such as gene expression analysis or targeted protease assays, would be necessary to validate these interactions. The chemically diverse composition of CEO constituents possibly acts synergistically, and their combined effects may contribute to the overall attenuation of *C. albicans* virulence.

In addition to its antivirulence activity, such as inhibition of germ tube formation, biofilm development, and secreted protease activity, CEO also exhibits immunomodulatory potential. This aspect was investigated through a macrophage co-culture model, wherein sub-MIC concentrations of CEO were used to assess alterations in phagocytic response, intracellular germ tube formation, and cytokine levels. CEO-treated *C. albicans* cells exhibited an inability to develop germ tubes, which is a key virulence factor that drives tissue invasion and immune activation. The loss of hyphal transition, a central determinant of *C. albicans* pathogenicity,^29,58^ may potentially influence downstream inflammatory signaling pathways.

Consistent with this, CEO treatment resulted in notable decreases in pro-inflammatory cytokines, including IL-6, IL-12, IL-23, TNF-α, and IL-1β, alongside an increase in the anti-inflammatory cytokine IL-10, indicating a transition toward an immunoregulatory macrophage response. Moreover, CEO treatment resulted in an enhancement of macrophage-associated adhered and internalized *C. albicans* compared with untreated controls. As extracellular yeast cells were removed by PBS washing prior to macrophage lysis, the recovered CFU likely represent fungal cells adhered to or internalized by macrophages. This observation may indicate an enhanced interaction of CEO-treated yeast with macrophages, possibly due to altered fungal surface properties or reduced hyphal escape.^59,60^ However, additional studies would be required to distinguish whether this effect reflects changes in adhesion, uptake, or intracellular survival. Together, these findings suggest a dual mechanism: CEO suppresses fungal virulence by preventing morphogenesis and simultaneously modulates host macrophage signaling toward anti-inflammatory pathways, resulting in a net outcome that is both antivirulent and immunoregulatory.

## 5 Conclusion

In conclusion, this study demonstrates that cumin essential oil (CEO) exhibits antivirulence activity against *C. albicans* at sub-MIC concentrations without significant effects on fungal growth. CEO reduced key virulence-associated traits, including biofilm formation, yeast-to-hyphal transition, and secreted protease activity, suggesting its potential to interfere with key aspects of fungal pathogenicity. The downregulation of *ALS3*, *HWP1*, and *HSP90* indicate that CEO may influence pathways associated with adhesion, morphogenesis, and virulence regulation. In particular, the observed decrease in *HSP90* expression and docking analysis suggest a possible involvement of Hsp90-associated pathways. CEO also reduced secreted protease activity and while docking analysis indicates possible interactions with Sap proteins, these findings remain preliminary and require further validation. Additionally, CEO treatment modulated macrophage–fungal interactions, as reflected by altered phagocytic responses and cytokine profiles, suggesting a potential immunomodulatory effect. However, further studies are needed to elucidate the underlying mechanisms and confirm these observations in more complex models. Overall, these findings highlight the potential of CEO as a candidate antivirulence agent against *C. albicans*, warranting further investigation.

## Supplementary Material



## Data Availability

The data underlying this study are available in the published article and its Supporting Information.

## Notes

Primer sequences used for real-time PCR; molecular docking analysis of CEO constituents with Hsp90, Sap4, Sap5, and Sap6 of *Candida albicans*, including docking scores and interaction residues; 2D interaction maps illustrating binding modes of major CEO constituents with target proteins; and GC–MS chromatogram of cumin essential oil (PDF)

## Abbreviations Full form

MTCC: microbial type culture collection
CFU: colony forming units
CEO: cumin essential oil
FBS: fetal bovine serum
MAPK: mitogen-activated protein kinase
PAMP: pathogen-associated molecular pattern
PMA: phorbol-12-myristate 13-acetate
PMN: polymorphonuclear cells
PRR: pattern recognition receptor
ROS: reactive oxygen species
RPMI: Roswell park memorial institute
RT-qPCR: reverse-transcription quantitative polymerase chain reaction
VVC: vulvovaginal candidiasis
PDA: PDB potato dextrose agar potato dextrose broth
AU: artificial urine
AT: artificial tear fluid
