# Supplementary material for: Antivirulence Strategy against Candida albicans: Cumin Essential Oil Attenuates Virulence and Enhances Host Immune Defense
Source: ACS Omega. 2026 May 14;11(20):29572–89. doi: 10.1021/acsomega.5c12490 (PMC13216983; doi:10.1021/acsomega.5c12490)
Supplement: Supplementary file 1 [file ao5c12490_si_001.pdf]

Anti-virulence strategy against *Candida albicans*: Cumin essential oil attenuates virulence and enhances host immune defence

Nimisha Mahesh[a], Afia Harith[a], Anu R Melge[a],
Attumpurathu Nandini[a], Aravind Madhavan[a], Kuniyil Abhinand[a],
Peralam Yegneswaran Prakash[b], Bipin G Nair[a], Geetha B Kumar[a], Jayalekshmi Haripriyan[a]*
[a]Amrita School of Biotechnology, Amrita Vishwa Vidyapeetham, Amritapuri, Clappana P O-690525, Kollam, Kerala, India
[b]Department of Microbiology, Kasturba Medical College, Manipal, Manipal Academy of Higher Education, Manipal, Karnataka, 576104, India
***Corresponding author**
Jayalekshmi Haripriyan, Amrita School of Biotechnology, Amrita Vishwa Vidyapeetham, Clappana P O-690525, Kollam, Kerala, India
Tel: +919746347138
Email: jayalekshmih@am.amrita.edu

Supplementary tables

Table S1: primer sequence used for the real -time PCR

Gene	Primer name	Sequence (5′ → 3′)
HWP1	*HWP1-F*	TCTACTGCTCCAGCCACTGA
	HWP1-R	CCAGCAGGAATTGTTTCCAT
ALS3	*ALS3-F*	AATGCTGTTTTGGGTTGGTC
	ALS3-R	TCACCTGCCTGAAATTGACA
RAS1	*RAS1-F*	AGAACCGGTGAAGGGTTTTT
	RAS1-R	GCCAATGCTAATCCATCTTGA
HSP90	*HSP90-F*	CGATGAATATGCCATGACT
	HSP90-R	TCCATAGCAGATTCTCCAG
18S rRNA	*18S rRNA-F*	CGATGGAAGTTTGAGGCAATA
	18S rRNA-R	CTCTCGGCCAAGGCTTATACT

Table S2: Molecular docking analysis of CEO constituents with Hsp90 of *Candida albicans.* Key amino acid residues involved in ligand-protein interactions within the Hsp90 binding sites are listed for each docked complex.

**Main Constituents**	**Docking Score** (kcal/mol)	**Interaction With Amino Acid Residues**
β-Pinene	-5.251 kcal/mol	LEU A:37, PHE A:127, LEU A:96, MET A:87, VAL A:139, LEU A:176
β-Myrcene	-5.358 kcal/mol	ILE A:80, ALA A:41, LEU A:37, VAL A:139, PHE A:127, LEU A:96, LEU A:176
α- Pinene	-4.853 kcal/mol	PHE A:127, VAL A:139, LEU A:176, MET A:87, LEU A:96

Table S3: Molecular docking analysis of CEO constituents with Sap4 of *Candida albicans.* Key amino acid residues involved in ligand-protein interactions within the Sap4 binding sites are listed for each docked complex

**Main Constituents**	**Docking Score** (kcal/mol)	**Interaction With Amino Acid Residues**
Cuminaldehyde	-5.803 kcal/mol	SER A:164, TYR A:160
γ-Terpinene	-5.699 kcal/mol	TYR A:160
β-Pinene	-5.554 kcal/mol	TYR A:160, ALA A:195, ARG A:196, ILE A:199, ILE A:106
ρ-Cymene	-5.71 kcal/mol	TYR A:160
β-Myrcene	-4.853 kcal/mol	ILE A:199, TYR A:160, ILE A:106, ARG A:196, ILE A:88
α-Phellandrene	-5.618 kcal/mol	TYR A:160, ILE A:199, ILE A:106
α- Pinene	-5.785 kcal/mol	ARG A:196, ALA A:195, ILE A:106, TYR A:160, ILE A:199

Table S4: Molecular docking analysis of CEO constituents with Sap5 of *Candida albicans.* Key amino acid residues involved in ligand-protein interactions within the Sap5 binding sites are listed for each docked complex.

Main Constituents	Docking Score (kcal/mol)	Interaction With Amino Acid Residues
γ-Terpinene	-5.614 kcal/mol	TYR A:160, ALA A:195, ILE A:199
β-Pinene	-5.585 kcal/mol	TYR A:160, ALA A:195, ILE A:199, ARG A:196, ILE A:106
ρ-Cymene	-5.645 kcal/mol	ILE A:199, ALA A:195, TYR A:160
α-Terpinen-7-al	-5.645 kcal/mol	SER A:164, TYR A:160
β-Myrcene	-4.71 kcal/mol	TYR A:160, TRP A:127, ARG A:196, ILE A:88, ILE A:106, ILE A:199
α-Phellandrene	-5.326 kcal/mol	LEU A:396, PHE A:204, ILE A:219
α- Pinene	-5.747 kcal/mol	ARG A:196, ALA A:195, TYR A:160, ILE A: 106, ILE A:199

Table S5: Molecular docking analysis of CEO constituents with Sap6 of *Candida albicans.* Key amino acid residues involved in ligand-protein interactions within the Sap6 binding sites are listed for each docked complex.

Main Constituents	Docking Score (kcal/mol)	Interaction With Amino Acid Residues
Cuminaldehyde	-5.178 kcal/mol	TYR A:357, LYS A:35, LEU A:359, TYR A:360
β-Pinene	-4.758 kcal/mol	TRP A:127, LYS A:35, ILE A:88
α-Terpinen-7-al	-5.221 kcal/mol	ARG A:128, LYS A:35, ILE A:88
γ-Terpinen-7-al	-5.188 kcal/mol	LYS A:35, LEU A: 359, TYR A:357
β-Myrcene	-4.691 kcal/mol	ARG A:36, TYR A:357, LYS A:35, LEU A:359, TYR A:360
α-Phellandrene	-5.014 kcal/mol	ILE A:88, VAL A:34, TRP A:127, ARG A: 128, LYS A:35
α- Pinene	-4.844 kcal/mol	TRP A:127, LYS A:35, PHE A:32, ILE A:88

Supplementary figures

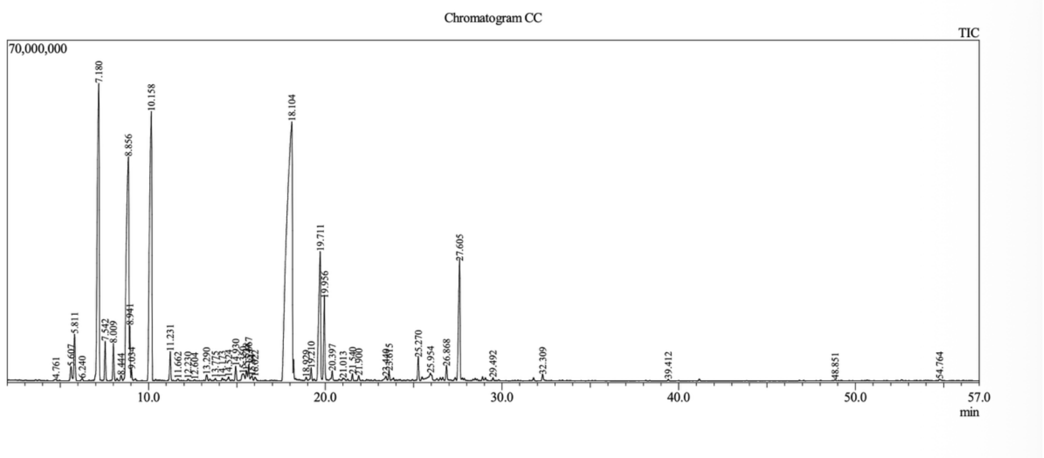

Figure S1. GC–MS analysis of cumin essential oil (CEO). The total ion chromatogram (TIC) depicts the volatile chemical profile of CEO, with major peaks corresponding to identified constituents.

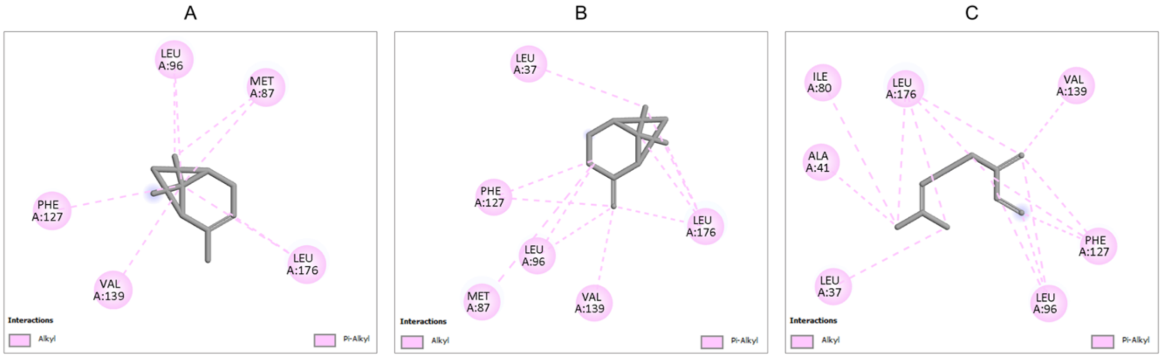

Figure S2. 2D interaction maps illustrating the binding modes of major constituents of CEO with *Candida albicans* Hsp90 obtained from molecular docking analysis. (A) α- Pinene, (B) β-Pinene and (C) Myrcene. The diagrams depict key intermolecular interactions, including hydrogen bonding, hydrophobic contacts, and π–alkyl interactions, between CEO constituents and amino acid residues within the Hsp90 binding pocket.

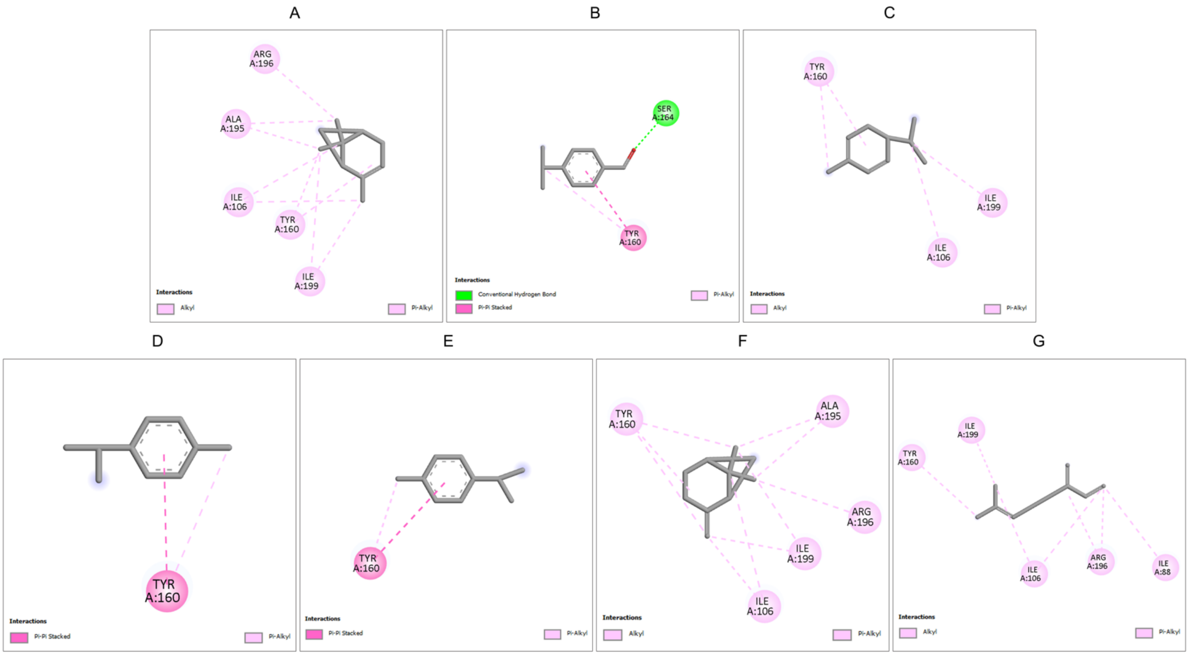

Figure S3. 2D interaction maps illustrating the binding modes of major constituents of CEO with *Candida albicans* Sap4 obtained from molecular docking analysis. (A) α- Pinene, (B) Cuminaldehyde, (C) α-Phellandrene, (D) γ-Terpinene, (E) ρ-Cymene, (F) β-Pinene and (G) Myrcene. The diagrams depict key intermolecular interactions, including hydrogen bonding, hydrophobic contacts, and π–alkyl interactions, between CEO constituents and amino acid residues within the Sap4 binding pocket.

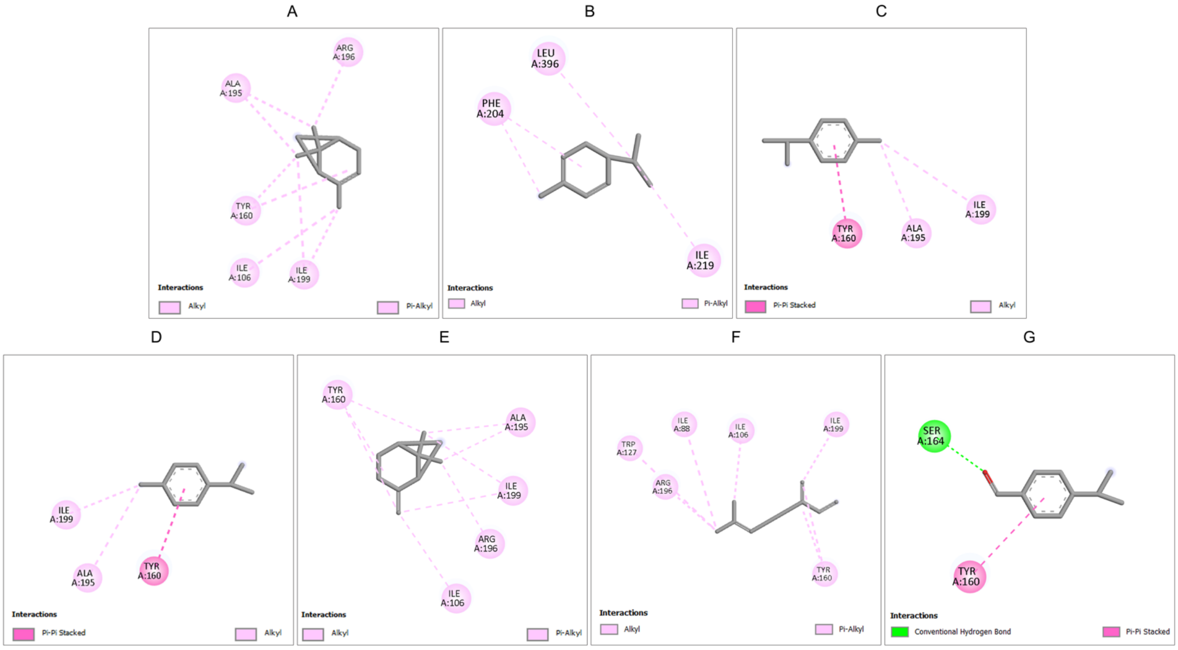

Figure S4. 2D interaction maps illustrating the binding modes of major constituents of CEO

with *Candida albicans* SAP5 obtained from molecular docking analysis. (A) α- Pinene, (B) α-Phellandrene, (C) γ-Terpinene, (D) ρ-Cymene, (E) β-Pinene, (F) Myrcene and (G) α-Terpinen-7-al. The diagrams depict key intermolecular interactions, including hydrogen bonding, hydrophobic contacts, and π–alkyl interactions, between CEO constituents and amino acid residues within the Sap5 binding pocket.

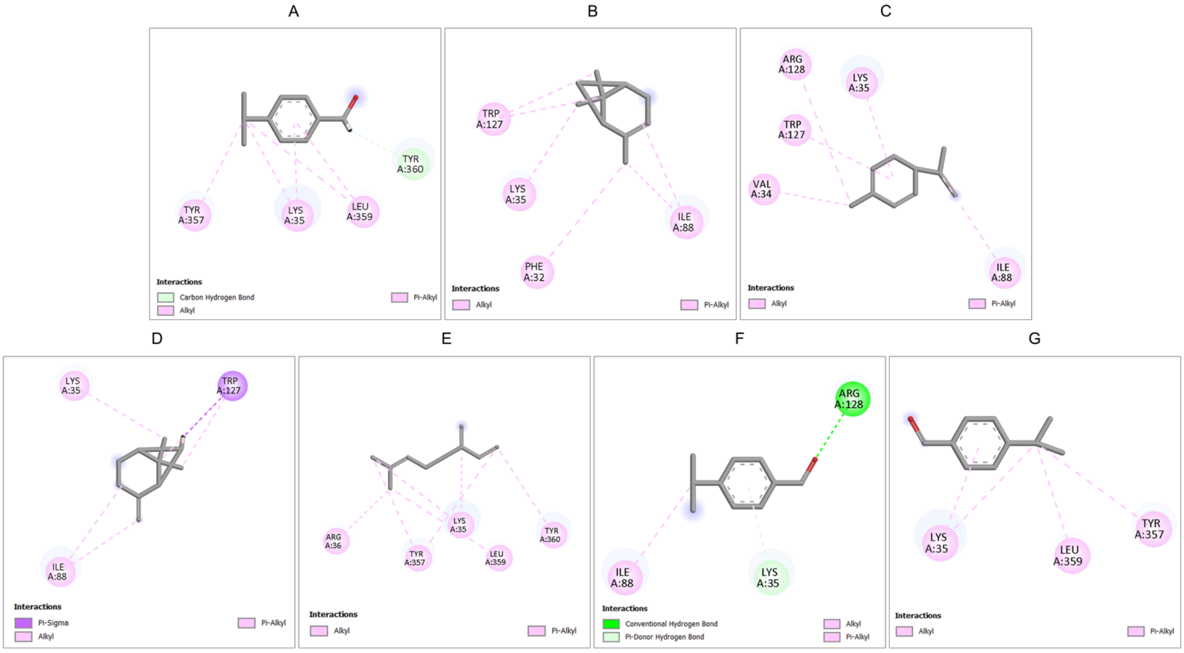

Figure S5. 2D interaction maps illustrating the binding modes of major constituents of CEO with *Candida albicans* SAP6 obtained from molecular docking analysis. (A) Cuminaldehyde, (B) α-Pinene, (C) α-Phellandrene, (D) β-Pinene, (E) Myrcene, (F) α-Terpinen-7-al and (G) γ-Terpinen-7-al. The diagrams depict key intermolecular interactions, including hydrogen bonding, hydrophobic contacts, and π–alkyl interactions, between CEO constituents and amino acid residues within the Sap6 binding pocket.
